# T lymphocyte SHP2-deficiency triggers anti-tumor immunity to inhibit colitis-associated cancer in mice

**DOI:** 10.18632/oncotarget.13812

**Published:** 2016-12-07

**Authors:** Wen Liu, Wenjie Guo, Lihong Shen, Zhen Chen, Qiong Luo, Xiaolin Luo, GenSheng Feng, Yongqian Shu, Yanhong Gu, Qiang Xu, Yang Sun

**Affiliations:** ^1^ State Key Laboratory of Pharmaceutical Biotechnology, School of Life Sciences, Nanjing University, Nanjing 210023, China; ^2^ Department of Medicine, University of California, San Diego, La Jolla, CA 92093, USA; ^3^ Department of Pathology, School of Medicine, University of California, San Diego, La Jolla, CA 92093, USA; ^4^ Department of Oncology, The First Affiliated Hospital of Nanjing Medical University, Nanjing 210029, China

**Keywords:** SHP2, colitis-associated cancer, cytotoxic T lymphocyte, IFN-γ, antitumor immunity

## Abstract

Nonresolving inflammation is involved in the initiation and progression process of tumorigenesis. Src homology 2 domain-containing tyrosine phosphatase 2 (SHP2) is known to inhibit acute inflammation but its role in chronic inflammation-associated cancer remains unclear. The role of SHP2 in T cells in dextran sulfate sodium (DSS)-induced colitis and azoxymethane-DSS-induced colitis-associated carcinogenesis was examined using SHP2^CD4−/−^ conditional knockout mice. SHP2 deficiency in T cells aggravated colitis with increased level of pro-inflammatory cytokines including IFN-γ and IL-17A. In contrast, the SHP2^CD4−/−^ mice developed much fewer and smaller tumors than wild type mice with higher level of IFN-γ and enhanced cytotoxicity of CD8^+^ T cells in the tumor and peritumoral areas. At the molecular level, STAT1 was hyper-phosphorylated in T cells lacking SHP2, which may account for the increased Th1 differentiation and IFN-γ secretion. IFN-γ neutralization or IFN-γ receptor knockout but not IL-17A neutralization, abrogated the anti-tumor effect of SHP2 knockout with lowered levels of perforin 1, FasL and granzyme B. Finally, the expression of granzyme B was negatively correlated with the malignancy of colon cancer in human patients. In conclusion, these findings suggest a new strategy to treat colitis-associated cancer via targeting SHP2.

## INTRODUCTION

The development of cancer is driven by various factors including genetic mutations [1] and epigenetic alterations [2]. In addition to many well established pathogenic processes, inflammation has emerged as a new hallmark of cancer which play an important role in initiation, progression, transformation, invasion and metastasis of cancer [3, 4]. Solid tumors are typically infiltrated with immunogenic cells, including T lymphocytes, macrophages and myeloid-derived suppressive cells. Specifically, CD4^+^ T cells are major players in orchestrating immune responses to tumor cells. On one hand, Th1 cells can secrete IFN-γ, which activates cytotoxic T lymphocytes (CTLs), together mediating anti-tumor immunity. On the other hand, Th1 cells also produce TNF-α and Th17 cells secrete IL-17, all of which may dampen immunity to tumor-associated antigens [5–7]. Depending on the subtypes of T cells and contexts of tumors, different molecular pathways and cellular processes may mediate distinct functional outcomes.

SHP2 is widely expressed in most tissues. As a signaling molecule, SHP2 plays vital roles in regulating diverse cellular processes including proliferation and differentiation [8, 9]. Aberrant regulation of SHP2 and its associated signaling pathways have been implicated in diseases such as cancer. Somatic mutations of SHP2-encoding *PTPN11* gene have been identified in patients with juvenile myelomonocytic leukemia, myelodysplastic syndrome, and acute myeloid leukemia [10, 11]. Activating mutation or overexpression of SHP2 may contribute to various types of cancer including gastric cancer [12], breast cancer [13] and glioma [14]. Therapeutically, SHP2 inhibitors has been developed to treat leukemia [15]. Although SHP2 was therefore regarded as a proto-oncogene based on these reports, recent studies demonstrated a decreased SHP2 expression in human hepatocellular carcinoma. Furthermore, loss of SHP2 in hepatocyte remarkably promoted carcinogenesis in mice [16]. These seemingly discrepancy in the role of SHP2 in cancer suggests that SHP2 may act as a tumor promoter or suppressor depending upon tissue/cell type.

Similar to its involvement in cancer, SHP2 may also critically regulate inflammation. A body of literature documented a negative role of this phosphatase in T cell activation [17, 18]. Interestingly, in CD4^+^ T lymphocytes, two major phosphatase targets of SHP2 are STAT1 (which triggers IFN-γ secretion) and STAT3 (which leads to production of IL-17A). Dephosphorylation of STAT1 and STAT3 by SHP2 results in their inactivation and inhibition of their downstream signal transduction. We therefore explored the role of SHP2 in these pathways, their functional relevance in immunity and the link to chronic inflammation-associated cancer.

In this study, we took advantage of mice with SHP2 deficiency in CD4^+^ T cells and two murine disease models, i.e., acute colitis and colitis-associated colon carcinoma. We demonstrate that SHP2 deficiency in T lymphocytes augments colitis but inhibits the development of colitis-associated colorectal cancer. In CD4^+^ T cells with SHP2 knockout, STAT1 is hyper-activated, resulting in increased Th1 differentiation and IFN-γ production. IFN-γ in turn enhances activity of CD8^+^ CTLs, i.e., anti-tumor immunity, which overrides the tumor-promoting microenvironment and suppresses tumor growth. Taken together, our findings define a novel strategy to promote anti-tumor immunity in colitis-associated colorectal carcinogenesis, specifically by targeting SHP2 to trigger IFN-γ signaling and the subsequent CTL activity.

## RESULTS

### SHP2 deficiency in CD4^+^ T cells aggravates inflammation and increases Th1 and Th17 cytokine profiles in DSS-induced colitis in mice

**Figure 1:**
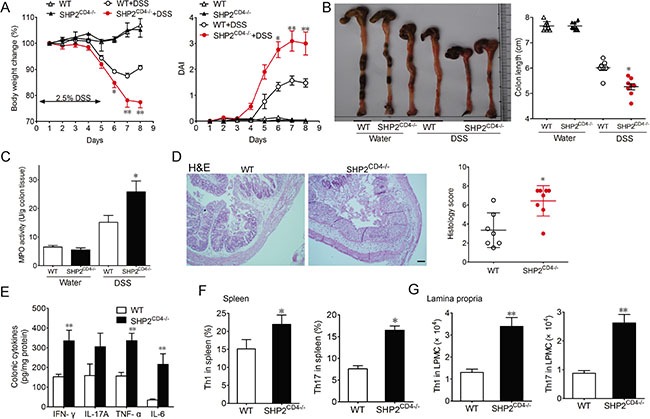
SHP2CD4−/− mice develop more severe colitis than WT mice in DSS-induced colitis model Mice were treated with 2.5% DSS in drinking water for 5 days to induce acute colitis. (**A**) Change of body weight is expressed as percentage of the original body weight. Disease activity index (DAI) was calculated by clinical scores indicated in Methods. (**B**) Gross anatomy of colons and colon length was measured. (**C**) Quantification of myeloperoxidase (MPO) activity in colonic tissues from WT vs SHP2^CD4−/−^ mice. (D, E) Hematoxylin and eosin (H&E)-staining (**D**) and ELISA of inflammatory cytokines (**E**) in colonic tissues from colitis mice on day 8. Scale bar, 2 mm. (**F**, **G**) Flow cytometry analysis of CD4^+^IFN-γ^+^ or CD4^+^IL-17A^+^ cells in the spleen or lamina propia isolated from colitis mice at day 8. Data are representative of three independent experiments (mean ± SEM of 8 mice per group). *P < 0.05, **P < 0.01 vs. WT group.

To determine the functional role of SHP2 in CD4^+^ T cells during the development of acute colitis, we compared between T-cell-conditional SHP2-knockout mice with their age- and sex-matched wild type (WT) littermates (Supplementary Figure S1). At the basal level, there was no significant difference in the counts of CD4^+^ and CD8^+^ T cells, neither in the CD4-CD8 ratio in the spleen or the lymph nodes between WT and SHP2^CD4−/−^ mice [19]. Next, we examined whether SHP2-deficiency in CD4^+^ T cells impacts the colitis progression using DSS-induced colitis model. Compared with the WT littermates, DSS induced severe colitis in SHP2^CD4−/−^ mice manifested by more body weight loss, higher disease activity index (DAI, a clinical parameter that reflects the severity of weight loss, rectal bleeding and stool consistency) and colon length shortening (Figure 1A, 1B). In line with these pathological symptoms, the biochemical and histological analysis also revealed more severe colon damage in SHP2^CD4−/−^ mice due to acute inflammation including higher MPO activity (a marker for neutrophil infiltration), more severe mucosal damage, necrosis and infiltration of inflammatory cells, i.e., neutrophils and monocytes (Figure 1C, 1D). In addition to these inflammatory markers, SHP2 deficiency in CD4^+^ T cells also lead to a more drastic loss of E-cadherin expression, evidence of deteriorated epithelium (Supplementary Figure S2).

We also examined the cytokine profile in the DSS-induced murine colitis which is similar to that of human with inflammatory bowel disease. Under DSS treatment, whereas WT mice already exhibited elevated levels of pro-inflammatory cytokines including IFN-γ, IL-17A, TNF-α and IL-6, SHP2^CD4−/−^ mice developed a profile with even higher levels of these pro-inflammatory cytokines (Figure 1E). Such profile is associated with an increased differentiation of T lymphocytes to Th1 and Th17 subtypes in the spleen (Figure 1F and 1G), whereas there was no significant difference in the differentiation of Th2 or Treg between the two groups of mice (Supplementary Figure S3). Taken together, results in Figure 1 indicate that mice lacking SHP2 in T cells are more susceptible to DSS-induced colitis.

### Loss of SHP2 in CD4^+^ T cells inhibits the development of CAC

**Figure 2:**
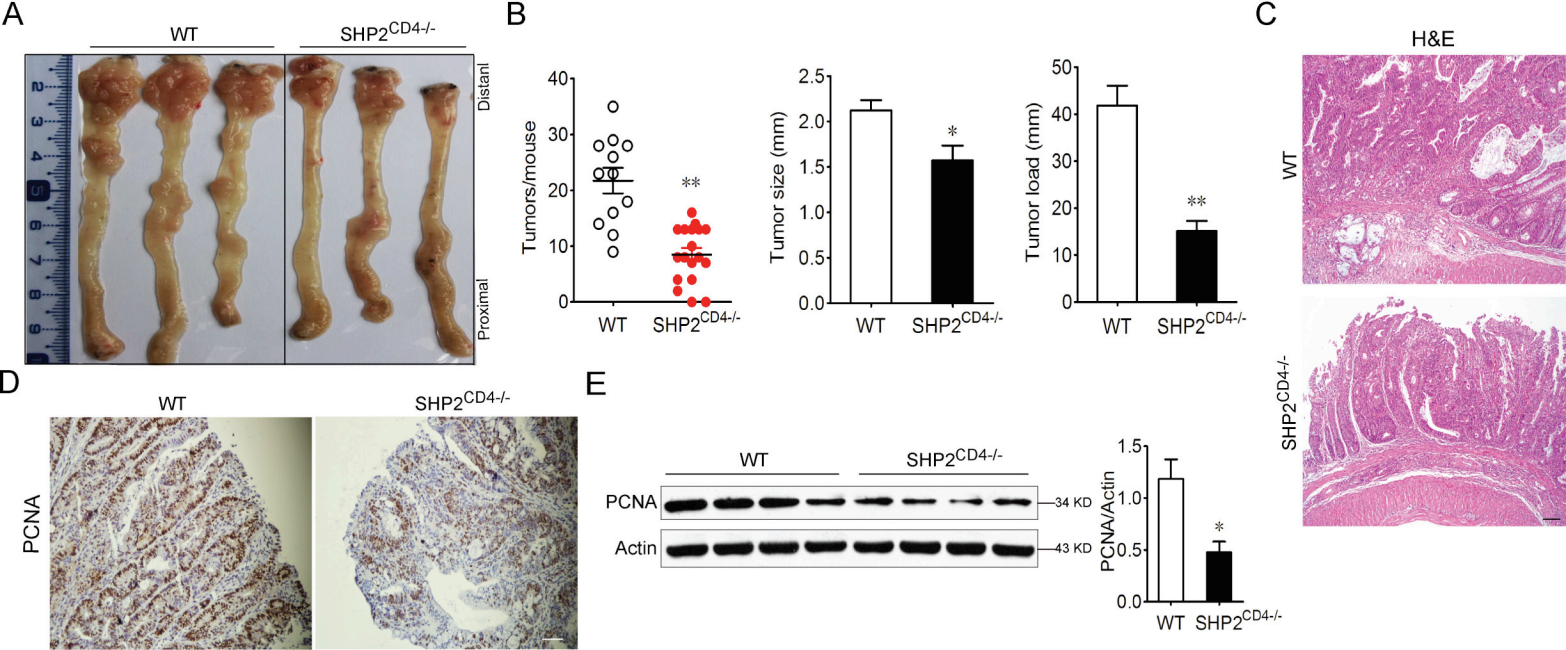
SHP2-deficiency leads to decreased development of colitis-associated colon (CAC) cancer WT and SHP2^CD4−/−^ mice were subjected to AOM-based induction protocol using three cycles of 2.5% DSS in the drinking water. (**A**) The colon was photographed. (**B**) The numbers, sizes and load of the tumors were measured. (**C**–**E**) H&E-staining in (C), IHC analysis of PCNA expression in (D), and immunoblotting of PCNA expression in (E) using colonic tissues from CAC mice on day 80. Data are representative of three independent experiments (mean ± SEM of 12 mice per group). Scale bar, 100 μm. *P < 0.05, **P < 0.01 vs. WT group.

To further examine whether more severe colitis observed in SHP2^CD4−/−^ mice also promotes colorectal carcinogenesis, we utilized a well-established model of CAC, i.e. AOM-DSS colitis-associated carcinoma model [20]. There was substantially more body weight loss in the SHP2^CD4−/−^ following each exposure to 2.5% DSS (Supplementary Figure S4), in accordance with DSS-induced acute colitis model. To our surprise, SHP2^CD4−/−^ mice developed less tumors than WT littermates, evidenced by the lower number, smaller size and lighter load of tumors (Figure 2A–2C). Moreover, the expression of proliferating cell nuclear antigen (PCNA) was profoundly decreased in SHP2^CD4−/−^ mice (Figure 2D, 2E). These results suggest that despite the augmented inflammation in these mice, colitis-associated tumorigenesis is attenuated.

### SHP2 deficiency in T cells results in a Th1 dominant tumor microenvironment and enhanced cytotoxicity of CD8^+^ T lymphocyte

**Figure 3:**
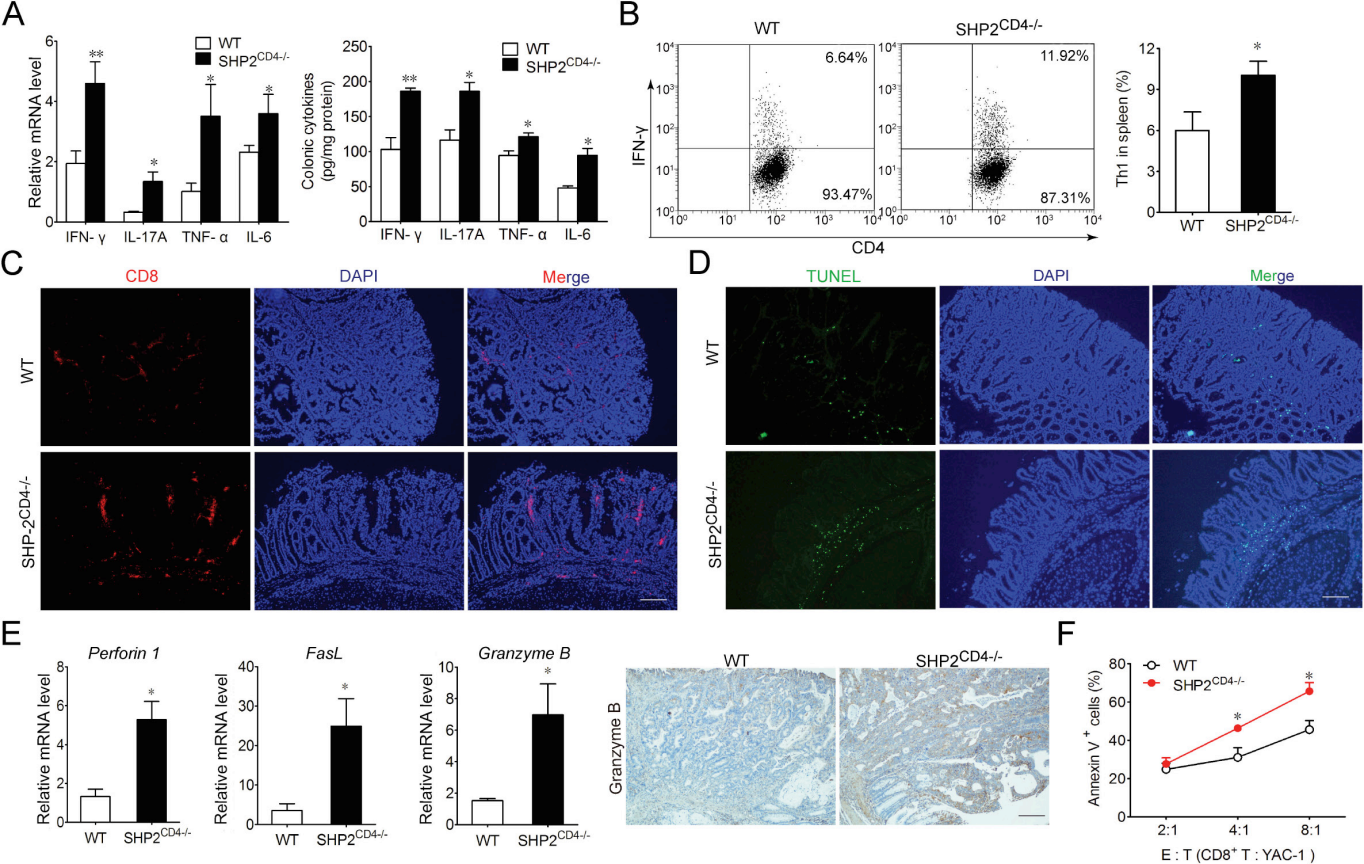
SHP2-deficiency results in a Th1 dominant tumor microenvironment with enhanced CD8+ CTL activity (**A**) Real-time PCR and ELISA of inflammatory cytokine expression on colonic tissues from CAC mice on day 80. (**B**) Flow cytometry analysis of CD4^+^IFN-γ^+^ cells in the spleen isolated from CAC mice at day 80 by intracellular staining. (**C**) Immunofluorescence imaging of CD8^+^ T cell infiltration in colonic tissues from CAC mice on day 80. (**D**) Apoptosis was analyzed by TUNEL staining in colonic tissues from CAC mice on day 80. (**E**) Real-time PCR analysis of *Perforin 1*, *Granzyme B*, and *FasL* or immunochemistry analysis of Granzyme B in colonic tissues from CAC mice on day 80. (**F**) Flow cytometry analysis of Annexin V^+^ YAC-1 cells (target cells, “T”) co-cultured with CD8^+^ cytotoxic T lymphocyte isolated from CAC mice (effector cells, “E”). Data are representative of three independent experiments (mean ± SEM of 12 mice per group). *P < 0.05, **P < 0.01 vs. WT group. Scale bar 100 μm.

To interrogate the mechanism underlying the aggravated inflammation and the unexpected attenuation of tumorigenesis in SHP2^CD4−/−^ mice, we analyzed the profile of pro-inflammatory cytokines including IFN-γ, IL-17A, TNF-α and IL-6 in colons of CAC mice. Similar to the observation in the colitis model (Figure 3A), compared to the WT littermates, SHP2^CD4−/−^ mice also developed persistently higher inflammatory level in the context of cancer. Furthermore, the differentiation to Th1 CD4^+^ T cell subtypes in SHP2^CD4−/−^ mice was increased when compared to WT littermates (Figure 3B). We next examined whether such Th1 dominant microenvironment in the SHP2^CD4−/−^ animals may contribute to the protection from tumorigenesis. Because the higher level of IFN-γ can promote CD8^+^ T cell-mediated cytotoxicity, we next examined the infiltration and activation of CD8^+^ T cells in the colons of SHP2^CD4−/−^ mice. Shown in Figure 3C and 3D, CD8^+^ T cell infiltration was increased in the colons of SHP2^CD4−/−^ animals, attendant with an increased tumor cell apoptosis. Accordingly, the expression levels of the cytotoxicity-related markers including perforin 1, granzyme B and FasL were significantly upregulated (Figure 3E). As shown in Figure 3F, CD8^+^ T cells isolated from SHP2^CD4−/−^ mice induced more YAC-1 cell death than that from WT mice, indicating higher CD8^+^ CTL activity in the KO mice. Collectively, these data suggest that the loss of SHP2 in CD4^+^ T cells promotes a prevalent Th1 immune response and CD8^+^ CTL activation.

### Loss of SHP2 promotes Th1 but not Th17 differentiation with an elevated IFN-γ/STAT1 signaling

**Figure 4:**
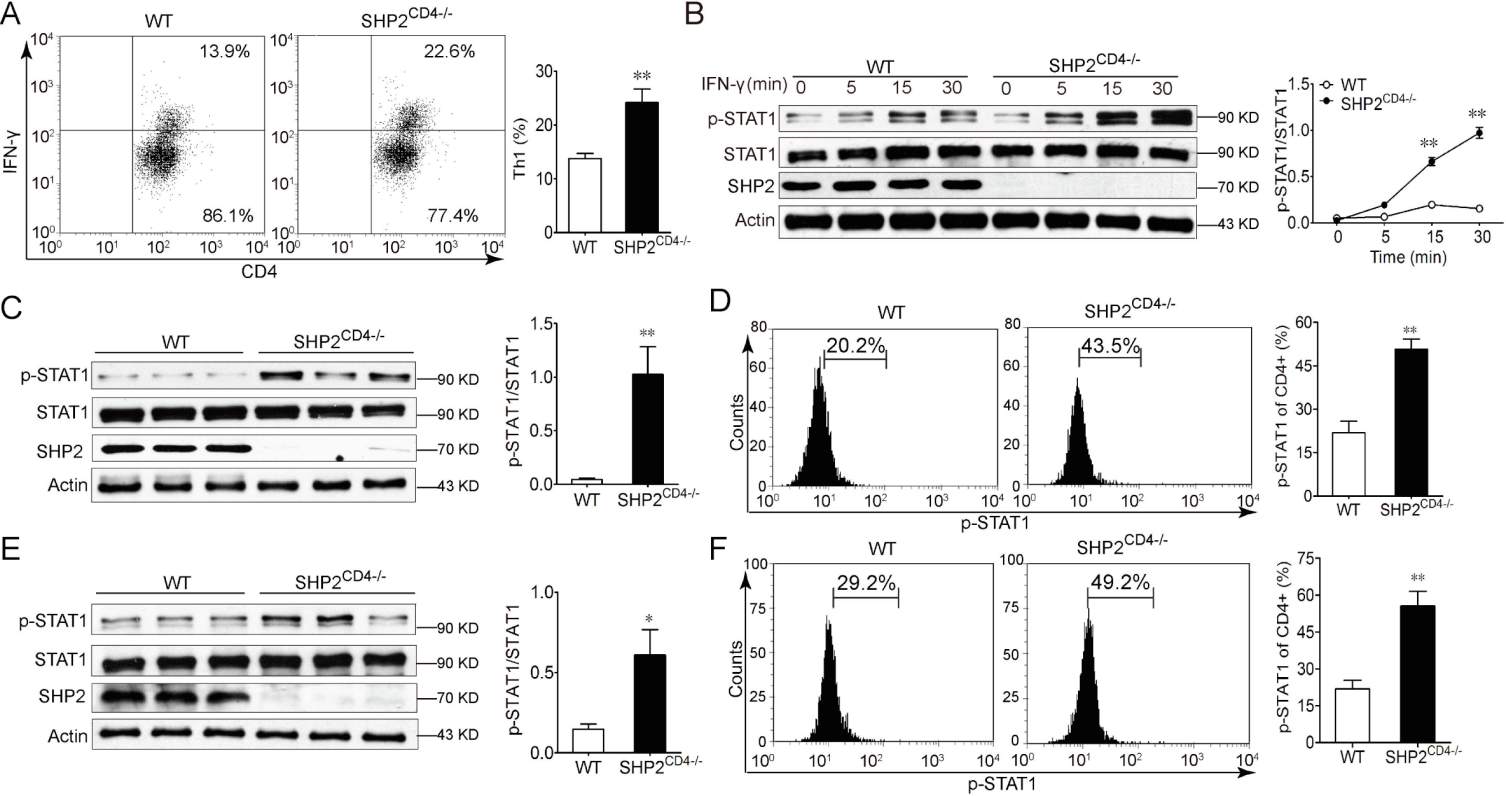
SHP2-deficiency in CD4+ T cells enhances IFN-γ-STAT1 signaling and Th1 differentiation (**A**) Flow cytometry analysis of CD4^+^IFN-γ^+^ cells in the spleen from WT and SHP2^CD4−/−^ mice. (**B**) Immunoblotting analysis of STAT1 activation in CD4^+^ T cells from WT and SHP2^CD4−/−^ mice in the presence of 20 ng/ml IFN-γ for indicated time. (**C**, **D**) Immunoblotting analysis (C) or flow cytometry analysis (D) of STAT1 activation in CD4^+^ T cells from mesenteric lymph node from DSS-induced colitis mice at day 8. (**E**, **F**) Immunoblotting analysis (E) or flow cytometry analysis (F) of STAT1 activation in CD4^+^ T cells from mesenteric lymph node from AOM-DSS-induced CAC mice at day 80. Data are representative of three independent experiments (mean ± SEM of 6 mice per group). *P < 0.05, **P < 0.01 vs. WT group.

To confirm the prevalence of the Th1 differentiation in SHP2^CD4−/−^ mice, we compared the cytokine profile in the CD4^+^ T cells in the KO vs WT littermates. This comparison demonstrated that SHP2 deficiency led to more Th1 cytokine but not Th17, Treg or Th2 cytokine secretion (Figure 4A, Supplementary Figures S3, S5, S6). To further delineate the molecular mechanism underlying the SHP2 deficiency and Th1 differentiation, we examined the STAT1 activation, downstream of IFN-γ in the CD4^+^ T cells. When stimulated with IFN-γ *in vitro*, the CD4^+^ T cells responded with an enhanced STAT1 phosphorylation in a time-dependent manner (Figure 4B). This increased STAT1 phosphorylation was recapitulated *in vivo*, i.e. in mesenteric lymph node T cells from mice with DSS-induced colitis or AOM-DSS-induced CAC (Figure 4C–4F). These findings suggest that in the T cells of WT animals, SHP2 is likely to inhibit Th1 differentiation via IFN-γ/STAT1 pathway.

### IFN-γ signaling is essential for mediating the cytotoxicity of CD8^+^ T lymphocyte and protection of SHP2^CD4−/−^ mice against CAC

**Figure 5:**
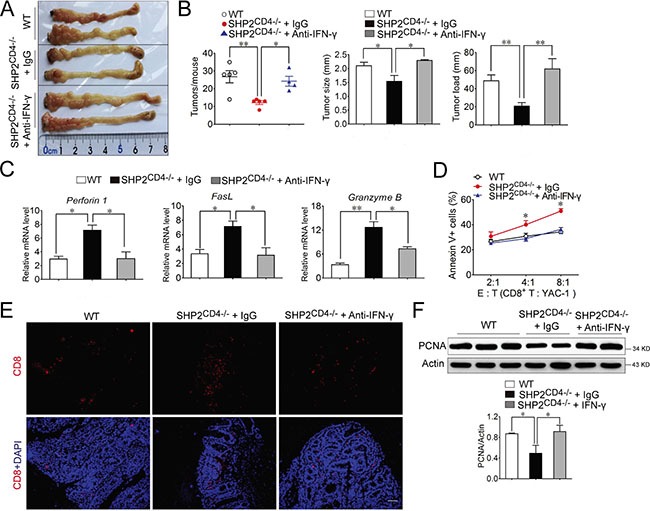
IFN-γ neutralization reverses the inhibitory effect of SHP2-deficiency on CAC Mice were injected intraperitoneally with 25 μg anti-IFN-γ neutralized antibody or IgG control every 4 days after the last DSS cycle. (**A**) The inside of the colon was photographed. (**B**) The numbers, sizes and load of the tumors were measured. (**C**) Real-time PCR analysis of perforin 1, granzyme B, and FasL expression in colonic tissues from CAC mice on day 80. (**D**) Flow cytometry analysis of Annexin V^+^ YAC-1 cells in the co-culture with CD8^+^ cytotoxic T lymphocyte isolated from CAC mice at day 80. Target (T) cells: YAC-1 ; Effector (**E**) cells: CD8^+^ T cells. *P < 0.05 vs. IgG group. (E) Immunofluorescence analysis of CD8^+^ T cell infiltration in colonic tissues from CAC mice on day 80. Scale bar, 100 μm. (**F**) Immunoblotting analysis of PCNA expression on colonic tissues from CAC mice on day 80. Data are representative of three independent experiments (mean ± SEM of 6 mice per group). *P < 0.05, **P < 0.01.

**Figure 6:**
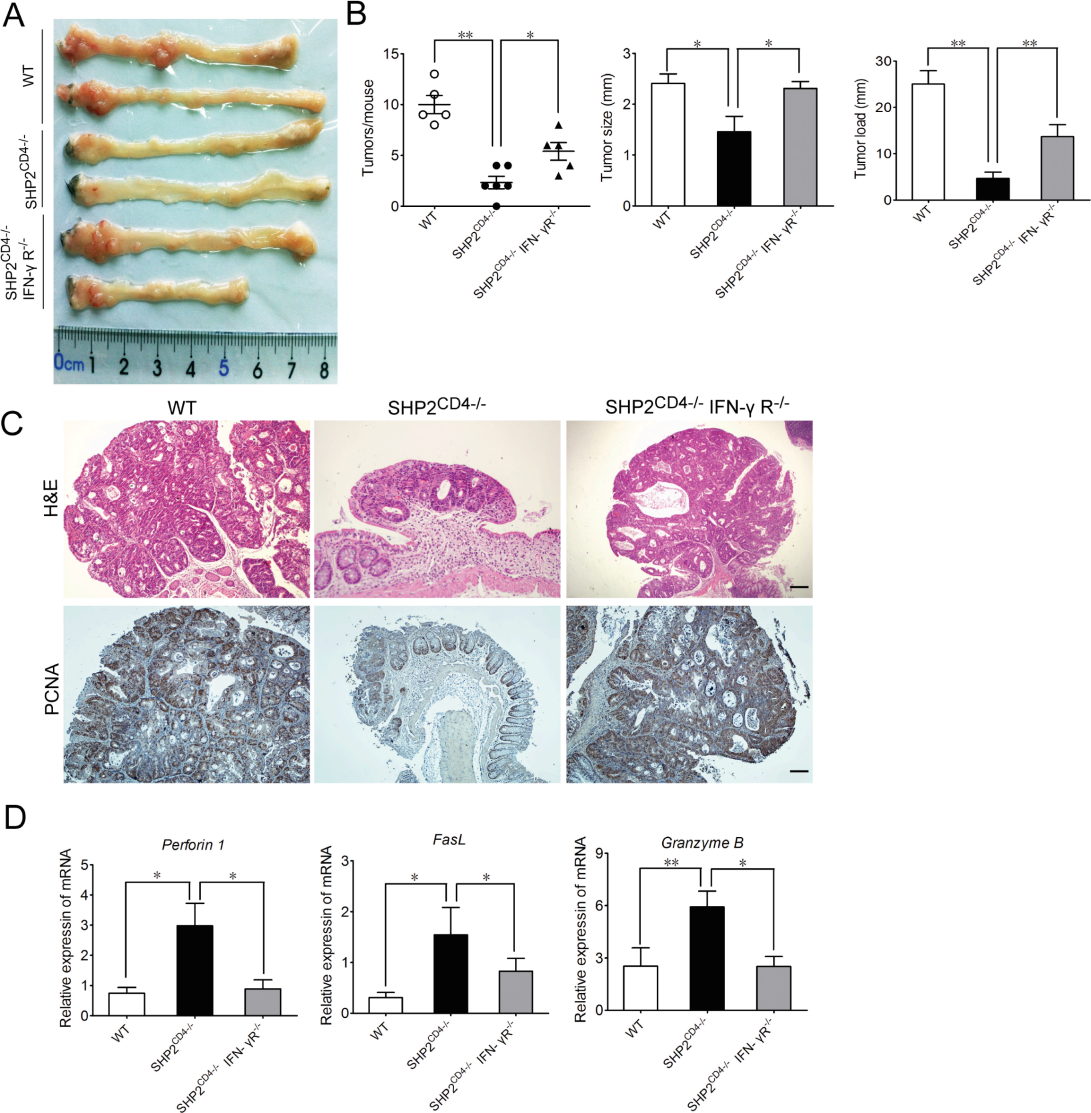
IFN-γ signaling is required for the inhibitory effect of SHP2-deficiency on CAC SHP2 and IFN-γR double knockout mice were breed and subjected to AOM-DSS model. (**A**) The inside of the colon was photographed. (**B**) The numbers, sizes and load of the tumors were measured. (**C**) Hematoxylin and eosin (H&E)-stained colonic tissue sections harvested from CAC mice on day 80. Immunochemistry analysis of PCNA expression on colonic tissue sections from CAC mice on day 80. Scale bar, 100 μm. (**D**) Real-time PCR analysis of Perforin 1, Granzyme B, and FasL in colonic tissues from CAC mice on day 80. Data are representative of three independent experiments (mean ± SEM of 5 mice per group in B, D). *P < 0.05, **P < 0.01.

To further establish the role of IFN-γ in the CAC progression in SHP2^CD4−/−^ mice, we perturbed IFN-γ signaling through antibody neutralization (as demonstrated in Supplementary Figure S7) and genetic ablation of IFN-γ receptor (IFN-γR), i.e., SHP2^CD4−/−^IFN-γR^−/−^. Shown in Figure 5A and 5B, the tumor incidence, including the number, the size and the load were almost completely restored to the level in WT mice when SHP2^CD4−/−^ mice were administered anti-IFN-γ antibody (Figure 5A, 5B). Similarly, SHP2^CD4−/−^IFN-γR^−/−^ mice developed comparable tumor as WT mice (Figure 6A, 6B). Consistently, the molecular alterations due to SHP2 deficiency in CD4^+^ T cells, e.g. PCNA expression, were largely reversed, if not all, through these *in vivo* perturbations (Figure 5C, Figure 6C, Supplementary Figure S8A, S8B,). In terms of the cytotoxicity of CD8^+^ T cells (also significantly enhanced in SHP2^CD4−/−^ mice), the anti-IFN-γ treatment markedly reduced CD8^+^ T cell infiltration in the colon tissues (Figure 5D), the cytotoxicity of the isolated CD8^+^ T cells (Figure 5E) and tumor cell apoptosis (Supplementary Figure S8C). Accordingly, the mRNA levels of the cytotoxicity-related markers Perforin 1, Granzyme B and FasL were also decreased by IFN-γ neutralization (Figure 5F). In parallel, these effects were also observed in SHP2^CD4−/−^IFN-γR^−/−^ mice when compared with SHP2^CD4−/−^ littermates (Figure 6D). Together, Figure 5 suggests that the IFN-γ signaling is essential for mediating the cytotoxicity of CD8^+^ T lymphocyte and protection of SHP2^CD4−/−^ mice against CAC.

### Granzyme B expression in human colorectal cancer is negatively correlated with the malignancy of colorectal cancer

**Figure 7:**
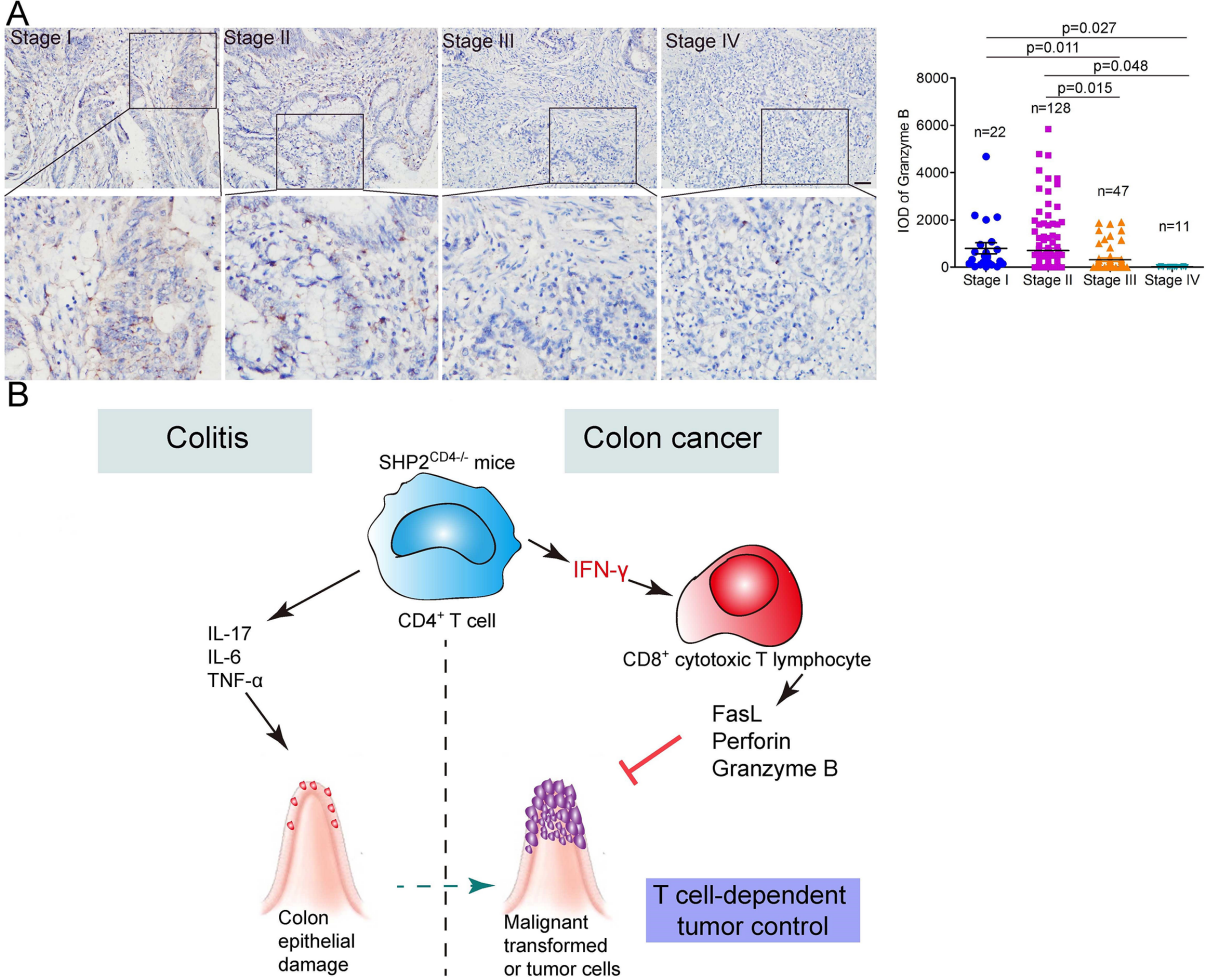
Anti-tumor immunity negatively correlates with the malignance of human colon cancer (**A**) Immunohistochemistry analysis of granzyme B expression in the tissue arrays of colon cancer tissues from patients with different clinical stages of carcinoma (I-IV). Representative images and the integrated optical intensity of different stages of carcinomas are shown. Data are presented as mean ± s.e.m. Scale bar, 100 μm. (**B**) Illustration for the mechanism of T cell SHP2-deficiency in colitis or CAC model. Loss of SHP2 in CD4^+^ T cells triggers over-production of inflammatory cytokines including IFN-γ and IL-17A. On the hand, IL-17A promotes colon epithelial damage and malignant transformation. On the other hand, IFN-γ enhances activity of CD8^+^ cytotoxic T lymphocyte, which evidenced by increased levels of perforin 1, FasL and granzyme B. CD8^+^ T cell-mediated anti-tumor immunity reverses tumor-promoting microenvironment and finally suppresses tumor growth.

To provide evidence supporting the association of IFN-γ signaling, CD8^+^ T cell function and the progression of colorectal cancer, we evaluated the protein levels of granzyme B in tissue arrays of colon cancer tissues from human patients with different clinical stages of carcinoma (stages I-V, Supplementary Table S1). Illustrated in Figure 7A, modest granzyme B expression was detected in stage I (*n* = 22) and stage II malignant tumor tissues (*n* = 128). In contrast, a significantly decreased granzyme B expression was detected in stage III (*n* = 47, *p* = 0.011 vs. stage I, *p* = 0.015 vs. stage II) and stages IV malignant tumor tissues (*n* = 11, *p* = 0.027 vs. stage I, *p* = 0.048 vs. stage II). These data from clinical samples suggest that a higher level of CD8^+^ CTL activity may be negatively associated with the severity and malignancy of colon cancer.

## DISCUSSION

It is now widely accepted that chronic nonresolving inflammation contributes to the initiation and progression of tumor development [21]. In patients with inflammatory bowel diseases such as ulcerative colitis and Crohn's disease, the risk of developing colorectal cancer is much higher [22–24]. In line with the previous findings suggesting the negative regulation of SHP2 in acute inflammation, our present study show the deletion of SHP2 in CD4^+^ T cells aggravates the DSS-induced colitis (Figure 1). However, when SHP2^CD4−/−^ mice were subjected to the AOM-DSS model of CAC, we observed an unexpected reduction of tumor incidence and progression compared with WT mice (Figure 2). This seemingly paradox prompted us to examine the pro-inflammatory response mediated by CD4^+^ T cells with SHP2 deficiency and its possible outcome in anti-tumor immunity.

CD4^+^ T lymphocytes are classified mainly into four subsets, namely, Th1, Th2 and Th17 or Treg according to their cytokine secretion. These four subsets play differential roles during tumor development. Th1 cells can repress tumor growth by secreting IFN-γ and supporting CTL function [25, 26]. Th2 cells generate IL-4 and IL-13 to promote tumor growth by stimulating tumor cell proliferation [27] or promoting tumor cell resistance to apoptosis [28]. Th17-derived IL-17 can stimulate STAT3 signaling [7] or promote myeloid-derived suppressor cell, both nurturing tumor microenvironments [29]. Treg cells foster tumor expansion by suppressing CD8^+^ CTLs and natural killer cells [30]. In line with previous findings from our group and others that SHP2 inhibits JAK-STAT1 signaling [18, 31, 32], we found that SHP2 knockout in CD4^+^ T lymphocyte resulted in a Th1-dominant differentiation, hallmarked by the robust induction of IFN-γ (Figure 3), hyper-phosphorylation of STAT1 (Figure 4) and the consequent strong activation of CD8^+^ CTLs (Figure 3). In contrast, SHP2-deficiency had no significant effect on Th2 or Treg cell differentiation (Supplementary Figure S3). Although IL-17A is increased in SHP2^C^D4−/− mice (Figure 1E, Figure 3A) and STAT3 is also hyper-phosphorylated in SHP2-deficient T cells (data not shown), SHP2 ablation had no significant influence on Th17 differentiation *in vitro* (Supplementary Figure S5). Taken together, the preferential differentiation of CD4^+^ T lymphocyte into the anti-tumor Th1 cells may contribute to the attenuated colon cancer mediated through SHP2 deficiency.

As the main cytokine secreted by Th1 cells, IFN-γ plays a pivotal role in inflammatory processes and anti-tumor immunity. This is supported by the previous study demonstrating that IFN-γ-deficient mice are more susceptible to inflammation-induced liver cancer [33]. The anti-tumor activity of IFN-γ can be partially explained by its capacity to trigger Fas-mediated target cell death or perforin-induced cytolysis, both of which are tumor-destroying processes of CD8^+^ CTLs [34]. In our current study, the increased IFN-γ seen in the SHP2-deficient CD4^+^ T cells led to a higher infiltration and cytotoxicity of CD8^+^ T cells, and further suppression of CAC. Indeed, both IFN-γ neutralization and IFN-γR knockout reversed this suppressive effect, corroborating the requirement of IFN-γ-dependent activation of CD8^+^ CTLs for protection against CAC in SHP2^CD4−/−^ mice. Since the level of IL-17A was also elevated in SHP2^CD4−/−^ mice than in WT mice (Figure 1E, Figure 3A), we examined the involvement of IL-17A in the tumorigenesis in SHP2^CD4−/−^ mice. As shown in Supplementary Figure S9B–S9D, opposite from the effect of IFN-γ neutralization, IL-17A neutralization decreased tumor number and load both in WT and SHP2^CD4−/−^ mice, suggesting that IL-17A may counteract the effect of IFN-γ to promote tumor, even though it is overridden by IFN-γ.

Based on this study, one may conclude that SHP2 is tumor-promoting. However, in our previous study using this same line of CD4^+^ T lymphocyte-specific SHP2 knockout (SHP2^CD4−/−^) mice, we found that SHP2 deletion promoted progression and metastasis of melanoma via IL-6-myeloid derived suppressor cells cascade [19]. Interestingly, the melanoma growth was first delayed in SHP2^CD4−/−^ mice, before the accelerated progression in the later stage. It is likely that during the onset or early stage of cancer, Th1-guided anti-tumor immunity dominates. However, in the advanced stages of cancer, chronic, tumor-promoting inflammation takes over. Collectively, these findings indicate that SHP2 may favor CD4^+^ T cell differentiation into distinct subtypes, depending on the stages and origins of different tumors.

Inflammation may be either pro-tumorigenic or anti-tumorigenic, depending on origin, duration, persistence and intensity [35]. The outcome of pro- or anti-tumorigenesis depends on the counter-balance among different immune cells, cytokines and regulating molecules. In this study, we provided evidence that in CD4^+^ T cells, SHP2 promotes the acute and persistent Th1-mediated immune response in colitis and CAC. Such process is characterized by high level of IFN-γ and activity of CTLs, both of which confer anti-tumor immunity to inhibit CAC (Figure 7B). These findings highlight a crucial role of SHP2-modulated T cell differentiation in CAC and may have clinical implications for CAC treatment.

## MATERIALS AND METHODS

### Mice

CD4^+^ T lymphocyte-specific SHP2 knockout mice (SHP2^CD4−/−^) were generated by crossing SHP2^flox/flox^ mice with CD4-Cre transgenic mice. A conditional mutant allele of SHP2 (SHP2 flox) was generated in mice using the Cre-loxP system by inserting two loxP sites into introns flanking exon 4, as described previously [36]. CD4-Cre transgenic mice was a gift from Prof. Zichun Hua in Nanjing University. SHP2^CD4−/−^ IFN-γR^−/−^ double knockout mice were generated by crossing SHP2^CD4−/−^ mice with IFN-γR^−/−^ knockout mice (JAX: B6.129S7-Ifngr^tm1Agt^/J). They were maintained with free access to pellet food and water in plastic cages at 21 ± 2 °C and kept on a 12 h light/dark cycle. Animal welfare was ensured and experimental procedures were carried out in accordance with the National Institutes of Health Guide for the Care and Use of Laboratory Animals, with the approval of the Animal Care and Use Committee of Nanjing University. All efforts were made to reduce the number of animals used and to minimize animals’ suffering.

### Chemicals, reagents and antibodies

Azoxymethane (AOM, A2853) and 4′, 6-diamidino-2-phenylindole (DAPI, D8417) were purchased from Sigma-Aldrich. Dextran sulfate sodium (DSS, 36–50 KD, 0216011080) was purchased from MP Biomedicals. Myeloperoxidase (MPO, A044) activity assay kit was purchased from Nanjing Jiancheng Bioengineering Institute. ELISA kits for murine TNF-α (12-2720), murine IL-17A (12-2170), murine IFN-γ (12-2000), murine IL-1β (12-2012) and human IL-1β (12-1012) were purchased from Beijing Dakewe Biotech. Cytokines including IL-12, IFN-γ, IL-6, IL-2 were purchased from R&D. Anti-IFN-γ and anti-IL-4 were from Peprotech. Anti-p-p65 (3033) was purchased from Cell Signaling Technology. Anti-actin (sc-1616), anti-COX2 (sc-19999) and anti-PCNA (sc-56) were purchased from Santa Cruz Biotechnology. GTVisin^TM^ anti-mouse/anti-rabbit immunohistochemistry analysis kit was purchased from Shanghai Gene Company.

### Induction and treatment of colitis-associated cancer and colitis

To induce colitis-associated cancer (CAC), mice were injected intraperitoneally (i.p.) with 7.5 mg/kg of AOM followed by three cycles of 2.5% DSS [20]. Mice were sacrificed on day 80 after CAC induction. Acute colitis was induced by giving 2.5% DSS in the drinking water for 7 days. The disease activity index was calculated as previously described [37]. Briefly, the following parameters were used for calculation: a) diarrhea (0 points = normal, 2 points = loose stools, 4 points = watery diarrhea); b) hematochezia (0 points = no bleeding, 2 points = slight bleeding, 4 points = gross bleeding).

### Histological analysis, immunohistochemistry and immunofluorescence

Human malignant colon adenocarcinoma tissue microarray (#CO2161) was purchased from US Biomax, Inc. Sections from murine colons were obtained for H&E staining and analyzed by a pathologist under light microscope (Olympus). For immunohistochemistry, immunofluorescence, or TUNEL staining, the sections were deparaffinized, rehydrated and washed in 1% PBS-Tween, and then treated with 2% hydrogen peroxide, blocked with 3% goat serum and incubated for 2 h at room temperature with specific primary antibodies. For TUNEL assay, the slides were stained with TUNEL-FITC (1:100) and then counter-stained with DAPI for 5 min. For immunohistochemistry, the slides were incubated with streptavidin-HRP for 40 min, then stained with DAB substrate and counter-stained with hematoxylin. Images were acquired by confocal laser-scanning microscope (Olympus FV1000). The integrated optical intensity of expression level in each sample was evaluated by Image-pro plus software.

### Real-time quantitative PCR

Real-time PCR was performed as reported previously [37], with primers detailed in the supplemental material.

### Western blot

The protocol for western blot has been reported previously [38].

### CD4^+^ T cell isolation and differentiation

CD4^+^ T cells were purified by magnetic cell sorting from spleen of wild-type (WT) and SHP2^CD4−/−^ mice and differentiated to Th1 with IL-12 (10 ng/ml) and IFN-γ (10 ng/ml), Th17 with IL-6 (20 ng/ml), TGF-β (1 ng/ml), anti-IFN-γ (10 μg/ml), and anti-IL-4 (1 μg/ml) for 72 h.

### Intracellular staining

The intracellular expression of IFN-γ or IL-17A in CD4^+^ T cells was analyzed using an eBioscience intracellular staining kit according to the manufacturer's instructions. Briefly, spleen cells from mice were incubated with phorbol 12-myristate 13-acetate (PMA, 100 ng/ml)/ionomycin (1 μg/ml) and monesine (1 μg/ml) in complete media at 37 °C for 6 h. Surface staining was performed with a CD4-FITC for 15 min at 4 °C. After this, the cells were fixed and permeabilized with fixation buffer and permeabilization wash buffer. The intracellular cytokine staining was performed with IL-17A-PE and IFN-γ-APC for 20 min. The cells were then analyzed by flow cytometry analysis.

### CD8^+^ T cell isolation and CTL assay

Cytotoxic activity was detected by flow cytometry-based cytotoxicity assay as described previously [39]. We then assessed the function of CD8^+^ T cells isolated from spleens of WT and SHP2^CD4−/−^ mice by co-culturing with YAC-1 cells (brief introduction of these cells). Cytotoxic activity is expressed as percentage of Annexin V positive target cells in the different groups.

### Antibody neutralization

Mice were injected intraperitoneally (i.p.) with IFN-γ neutralized antibody (R&D: 25 μg/mouse) or control IgG every 4 days after the last DSS cycle.

### Statistical analysis

Data are expressed as mean ± SEM. Student's *t*-test and one-way ANOVA test were used for comparison between two or more groups. *P* values of <0.05 or <0.01 were considered statistically significant.

## SUPPLEMENTARY MATERIALS FIGURES AND TABLE





## References

[1] Fearon ER (2011). Molecular genetics of colorectal cancer. Annu Rev Pathol.

[2] Pancione M, Remo A, Colantuoni V (2012). Genetic and epigenetic events generate multiple pathways in colorectal cancer progression. Patholog Res Int.

[3] Grivennikov SI, Karin M (2010). Inflammation and oncogenesis: a vicious connection. Curr Opin Genet Dev.

[4] Hanahan D, Weinberg Robert A (2011). Hallmarks of Cancer: The Next Generation. Cell.

[5] Zitvogel L, Tesniere A, Kroemer G (2006). Cancer despite immunosurveillance: immunoselection and immunosubversion. Nat Rev Immunol.

[6] Wang HY, Wang RF (2007). Regulatory T cells and cancer. Curr Opin Immunol.

[7] Wang L, Yi T, Kortylewski M, Pardoll DM, Zeng D, Yu H (2009). IL-17 can promote tumor growth through an IL-6-Stat3 signaling pathway. J Exp Med.

[8] Bennett AM, Hausdorff SF, O’Reilly AM, Freeman RM, Neel BG (1996). Multiple requirements for SHPTP2 in epidermal growth factor-mediated cell cycle progression. Mol Cell Biol.

[9] Neel BG, Gu H, Pao L (2003). The ‘Shp’ing news: SH2 domain-containing tyrosine phosphatases in cell signaling. Trends Biochem Sci.

[10] Konieczna I, Horvath E, Wang H, Lindsey S, Saberwal G, Bei L, Huang W, Platanias L, Eklund EA (2008). Constitutive activation of SHP2 in mice cooperates with ICSBP deficiency to accelerate progression to acute myeloid leukemia. J Clin Invest.

[11] Tartaglia M, Niemeyer CM, Fragale A, Song X, Buechner J, Jung A, Hahlen K, Hasle H, Licht JD, Gelb BD (2003). Somatic mutations in PTPN11 in juvenile myelomonocytic leukemia, myelodysplastic syndromes and acute myeloid leukemia. Nat Genet.

[12] Dong S, Li FQ, Zhang Q, Lv KZ, Yang HL, Gao Y, Yu JR (2012). Expression and clinical significance of SHP2 in gastric cancer. J Int Med Res.

[13] Aceto N, Sausgruber N, Brinkhaus H, Gaidatzis D, Martiny-Baron G, Mazzarol G, Confalonieri S, Quarto M, Hu G, Balwierz PJ, Pachkov M, Elledge SJ, van Nimwegen E (2012). Tyrosine phosphatase SHP2 promotes breast cancer progression and maintains tumor-initiating cells via activation of key transcription factors and a positive feedback signaling loop. Nat Med.

[14] Liu KW, Feng HZ, Bachoo R, Kazlauskas A, Smith EM, Symes K, Hamilton RL, Nagane M, Nishikawa R, Hu B, Cheng SY (2011). SHP-2/PTPN11 mediates gliomagenesis driven by PDGFRA and INK4A/ARF aberrations in mice and humans. J Clin Invest.

[15] Hellmuth K, Grosskopf S, Lum CT, Wurtele M, Roder N, von Kries JP, Rosario M, Rademann J, Birchmeier W (2008). Specific inhibitors of the protein tyrosine phosphatase Shp2 identified by high-throughput docking. Proc Natl Acad Sci USA.

[16] Bard-Chapeau EA, Li S, Ding J, Zhang SS, Zhu HH, Princen F, Fang DD, Han T, Bailly-Maitre B, Poli V, Varki NM, Wang H, Feng GS (2011). Ptpn11/Shp2 acts as a tumor suppressor in hepatocellular carcinogenesis. Cancer Cell.

[17] Salmond RJ, Huyer G, Kotsoni A, Clements L, Alexander DR (2005). The src homology 2 domain-containing tyrosine phosphatase 2 regulates primary T-dependent immune responses and Th cell differentiation. J Immunol.

[18] You M, Yu DH, Feng GS (1999). Shp-2 tyrosine phosphatase functions as a negative regulator of the interferon-stimulated Jak/STAT pathway. Mol Cell Biol.

[19] Zhang T, Guo WJ, Yang Y, Liu W, Guo LL, Gu YH, Shu YQ, Wang L, Wu XF, Hua ZC, Ke YH, Sun Y, Shen Y (2013). Loss of SHP-2 activity in CD4(+) T cells promotes melanoma progression and metastasis. Sci Rep.

[20] Wirtz S, Neufert C, Weigmann B, Neurath MF (2007). Chemically induced mouse models of intestinal inflammation. Nat Protoc.

[21] Nathan C, Ding A (2010). Nonresolving inflammation. Cell.

[22] Terzic J, Grivennikov S, Karin E, Karin M (2010). Inflammation and Colon Cancer. Gastroenterology.

[23] Eaden JA, Abrams KR, Mayberry JF (2001). The risk of colorectal cancer in ulcerative colitis: a meta-analysis. Gut.

[24] Jess T, Loftus EV, Velayos FS, Harmsen WS, Zinsmeister AR, Smyrk TC, Schleck CD, Tremaine WJ, Melton LJ, P 3rd Munkholm, Sandborn WJ (2006). Risk of intestinal cancer in inflammatory bowel disease: a population-based study from olmsted county. Minnesota. Gastroenterology.

[25] Kaplan DH, Shankaran V, Dighe AS, Stockert E, Aguet M, Old LJ, Schreiber RD (1998). Demonstration of an interferon gamma-dependent tumor surveillance system in immunocompetent mice. Proc Natl Acad Sci USA.

[26] Dunn GP, Koebel CM, Schreiber RD (2006). Interferons, immunity and cancer immunoediting. Nat Rev Immunol.

[27] Aspord C, Pedroza-Gonzalez A, Gallegos M, Tindle S, Burton EC, Su D, Marches F, Banchereau J, Palucka AK (2007). Breast cancer instructs dendritic cells to prime interleukin 13-secreting CD4+ T cells that facilitate tumor development. J Exp Med.

[28] Todaro M, Lombardo Y, Francipane MG, Alea MP, Cammareri P, Iovino F, AB Di Stefano, C Di Bernardo, Agrusa A, Condorelli G, Walczak H, Stassi G (2008). Apoptosis resistance in epithelial tumors is mediated by tumor-cell-derived interleukin-4. Cell Death Differ.

[29] He D, Li H, Yusuf N, Elmets CA, Li J, Mountz JD, Xu H (2010). IL-17 promotes tumor development through the induction of tumor promoting microenvironments at tumor sites and myeloid-derived suppressor cells. J Immunol.

[30] Trzonkowski P, Szmit E, Mysliwska J, Mysliwski A (2006). CD4+CD25+ T regulatory cells inhibit cytotoxic activity of CTL and NK cells in humans-impact of immunosenescence. Clin Immunol.

[31] Wu X, Guo W, Wu L, Gu Y, Gu L, Xu S, Shen Y, Ke Y, Tan R, Sun Y, Xu Q (2012). Selective sequestration of STAT1 in the cytoplasm via phosphorylated SHP-2 ameliorates murine experimental colitis. J Immunol.

[32] Leibowitz MS, Srivastava RM, PA Andrade Filho, Egloff AM, Wang L, Seethala RR, Ferrone S, Ferris RL (2013). SHP2 is overexpressed and inhibits pSTAT1-mediated APM component expression, T-cell attracting chemokine secretion, and CTL recognition in head and neck cancer cells. Clin Cancer Res.

[33] Meng Z, Wang X, Gan Y, Zhang Y, Zhou H, Ness CV, Wu J, Lou G, Yu H, He C, Xu R, Huang W (2012). Deletion of IFNgamma enhances hepatocarcinogenesis in FXR knockout mice. J Hepatol.

[34] Listopad JJ, Kammertoens T, Anders K, Silkenstedt B, Willimsky G, Schmidt K, Kuehl AA, Loddenkemper C, Blankenstein T (2013). Fas expression by tumor stroma is required for cancer eradication. Proc Natl Acad Sci USA.

[35] Grivennikov SI, Greten FR, Karin M (2010). Immunity, inflammation, and cancer. Cell.

[36] Zhang EE, Chapeau E, Hagihara K, Feng GS (2004). Neuronal Shp2 tyrosine phosphatase controls energy balance and metabolism. Proc Natl Acad Sci USA.

[37] Guo W, Sun Y, Liu W, Wu X, Guo L, Cai P, Shen Y, Shu Y, Gu Y, Xu Q (2014). Small molecule-driven mitophagy-mediated NLRP3 inflammasome inhibition is responsible for the prevention of colitis-associated cancer. Autophagy.

[38] Guo WJ, Zhang YM, Zhang L, Huang B, Tao FF, Chen W, Guo ZJ, Xu Q, Sun Y (2013). Novel monofunctional platinum (II) complex Mono-Pt induces apoptosis-independent autophagic cell death in human ovarian carcinoma cells, distinct from cisplatin. Autophagy.

[39] Marcusson-Stahl M, Cederbrant K (2003). A flow-cytometric NK-cytotoxicity assay adapted for use in rat repeated dose toxicity studies. Toxicology.

