# Supplementary material for: T lymphocyte SHP2-deficiency triggers anti-tumor immunity to inhibit colitis-associated cancer in mice
Source: Oncotarget. 2016 Dec 7;8(5):7586–97. doi: 10.18632/oncotarget.13812 (PMC5352345; doi:10.18632/oncotarget.13812)
Supplement: Supplementary file 1 [file oncotarget-08-7586-s001.pdf]

T lymphocyte SHP2-deficiency triggers anti-tumor immunity to inhibit colitis-associated cancer in mice

Supplementary Materials

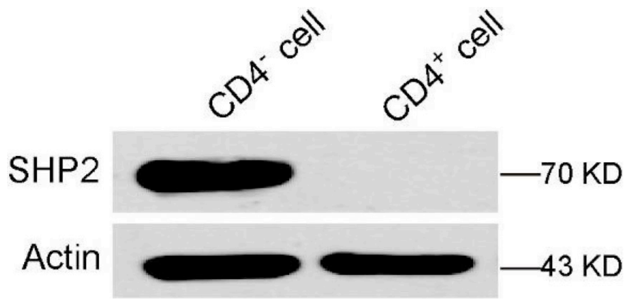

Supplementary Figure S1: CD4$^+$ T cell-specific knockout of SHP2 in SHP2CD4$^{-/-}$ mice. Immunoblotting analysis of SHP2 expression on CD4$^+$ T cells purified from lymph node of SHP2$^{CD4-/-}$ mice. Data are representative of three independent experiments.

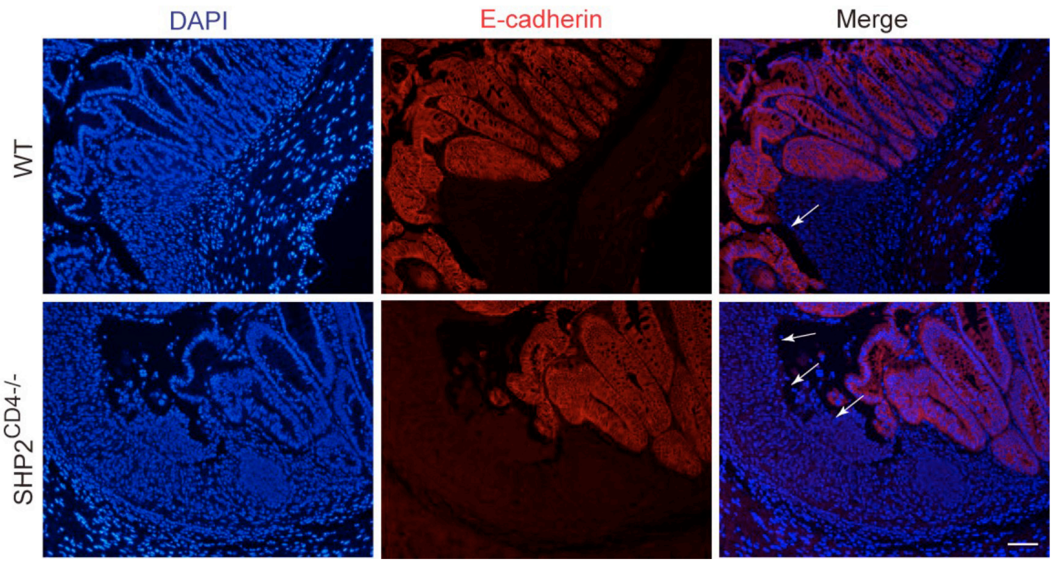

Supplementary Figure S2: SHP2 deficiency leads to more loss of epithelial E-cadherin in DSS-induced colitis model. Mice were treated with 2.5% DSS in drinking water for 5 days to induce acute colitis. Immunofluorescence analysis of E-cadherin expression in colonic tissues from colitis mice on day 8. Arrows indicate the area where E-cadherin is lost. Scale bar: 100 µm.

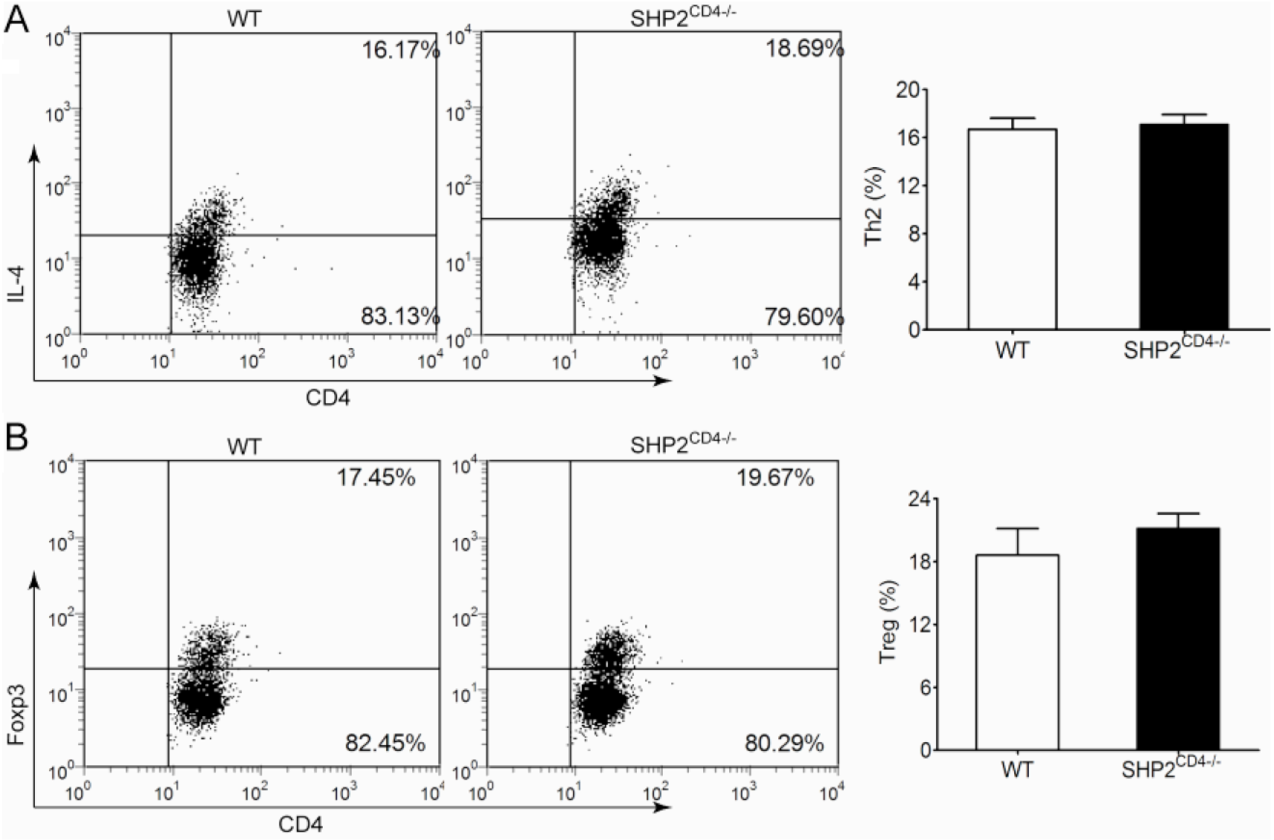

Supplementary Figure S3: SHP2-deficiency has no effect on Th2 or Treg cell differentiation in DSS-induced colitis model. Flow cytometry analysis of $CD4^+IL\text{-}4^+$ (Th2) and $CD4^+Foxp3^+$ (Treg) cells in T cells isolated from colitis mice at day 8. $CD4^+$ T cells were purified from mice with MicroBeads and differentiated *in vitro*. For Th2 or Treg differentiation, $CD4^+$ T cells were incubated with plate-bound mAbs of anti-CD3 and anti-CD28 under Th2 conditions (10 ng/ml IL-4, 10 µg/ml anti-IFN-γ mAb, 1 µg/ml anti-IL-12 mAb), or Treg conditions (1 ng/ml TGF-β, 20 ng/ml IL-2) for 72 h. Differentiated Th cells were washed and restimulated with plate-bound anti-CD3 mAb for 24 h, and cell were used for measuring cytokine levels by intracellular staining. Data are representative of three independent experiments (mean ± SEM of three independent samples).

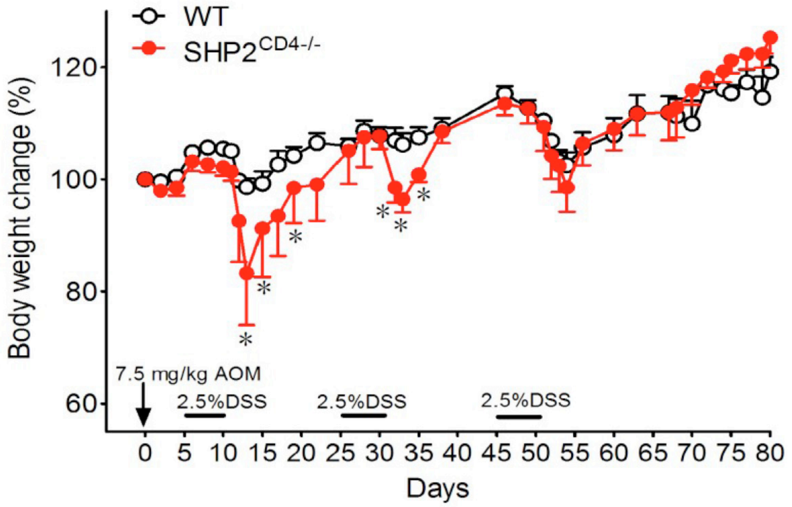

Supplementary Figure S4: SHP2$^{CD4-/-}$ mice exhibit significantly more body weight loss than WT mice in AOM-DSS model. WT and SHP2$^{CD4-/-}$ mice were subjected to AOM-based induction protocol using three cycles of 2.5% DSS in the drinking water. Body weight was recorded every day. Data are mean ± SEM of 12 mice per group. **P* < 0.05 vs. WT group.

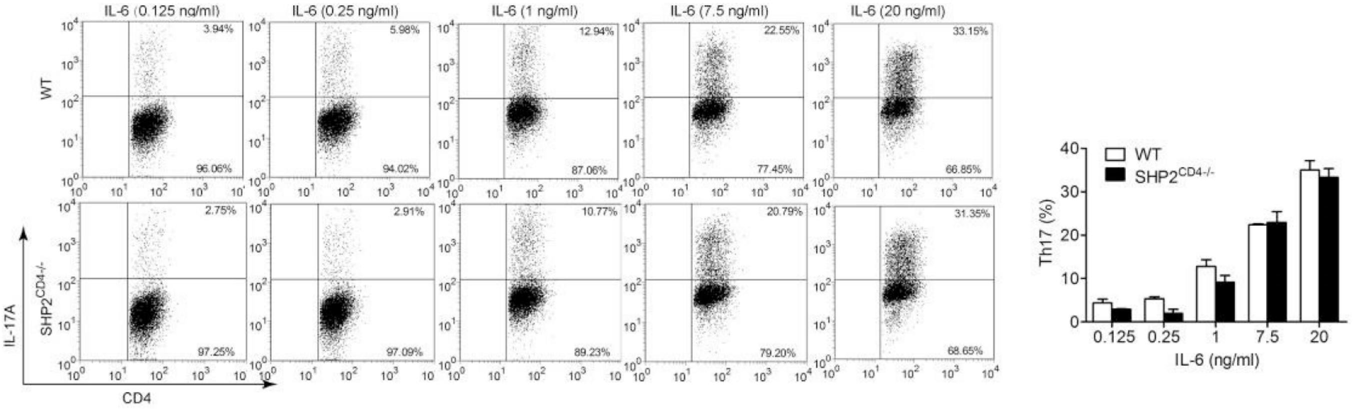

Supplementary Figure S5: SHP2-deficiency does not affect Th17 differentiation *in vitro*. Flow cytometry analysis of CD4^{+}IL-17A^{+} (Th17) in T cells isolated from WT and SHP2$^{CD4-/-}$ mice. Naive CD4^{+} T cells were purified from mice with MicroBeads and differentiated *in vitro*. For Th17 differentiation, naive CD4^{+} T cells were incubated with plate-bound mAbs of anti-CD3 and anti-CD28 under Th17 conditions (1 µg/ml anti-IL-4 mAb, 10 µg/ml anti-IFN-γ mAb, 20 ng/ml IL-6, 1 ng/ml TGF-β). Differentiated Th cells were washed and restimulated with plate-bound anti-CD3 mAb for 24 h, and cell were used for measuring cytokine levels by intracellular staining. Data are representative of three independent experiments (mean ± SEM of three independent samples).

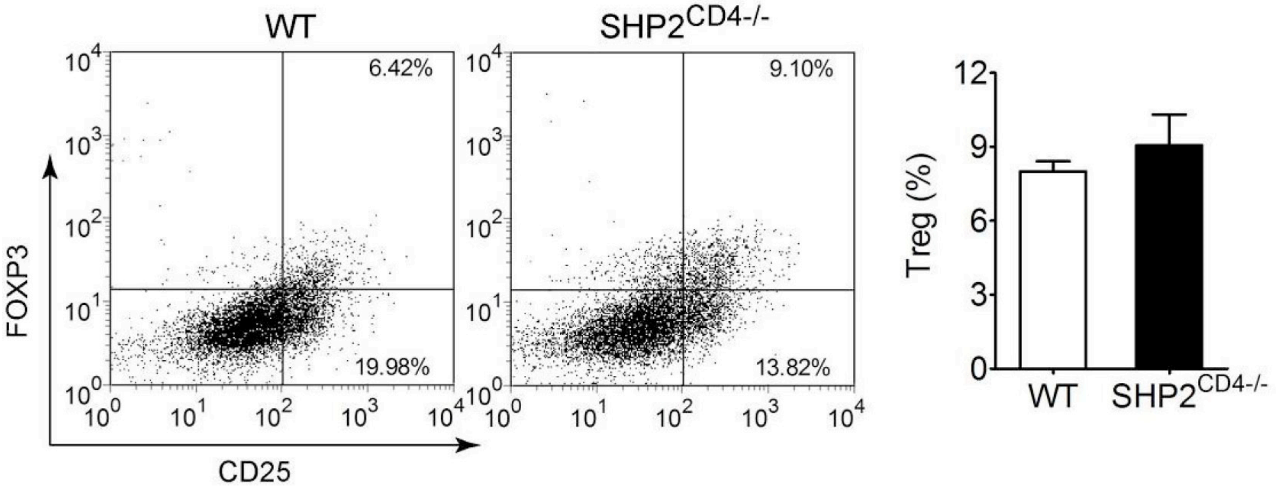

Supplementary Figure S6: Loss of SHP2 does not affect Treg differention *in vitro*. Flow cytometry analysis of CD4$^+$Foxp3$^+$ (Treg) cells in T cells isolated from WT and SHP2$^{CD4-/-}$ mice. Naive CD4$^+$ T cells were purified from mice with MicroBeads and differentiated *in vitro*. For Treg differentiation, naive CD4$^+$ T cells were incubated with plate-bound mAbs of anti-CD3 and anti-CD28 under Treg conditions (1 ng/ml TGF-β, 20 ng/ml IL-2) for 72 h. Differentiated Th cells were washed and restimulated with plate-bound anti-CD3 mAb for 24 h, and cell were used for measuring cytokine levels by intracellular staining. Data are representative of three independent experiments (mean ± SEM of three independent samples).

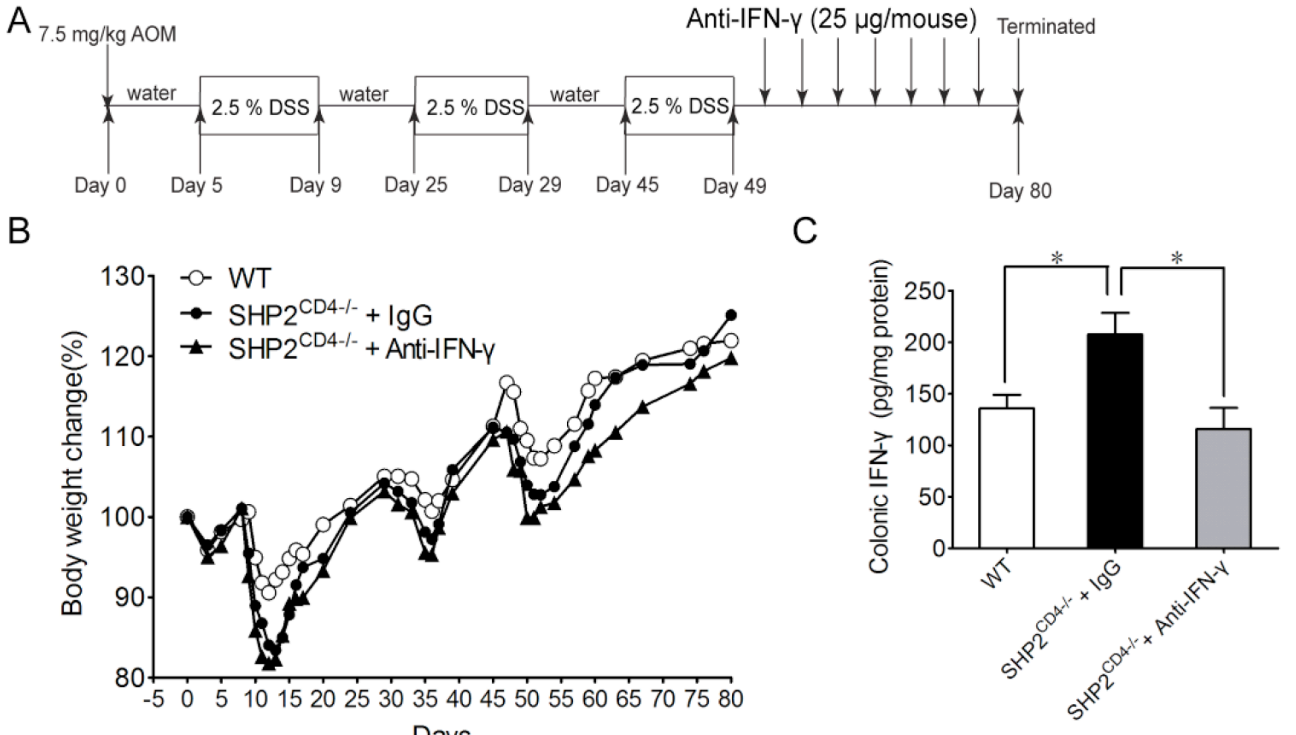

Supplementary Figure S7: The level of IFN-γ is suppressed by anti-IFN-γ antibody neutralization. WT and SHP2$^{CD4-/-}$ mice were subjected to AOM-based induction protocol using three cycles of 2.5% DSS in the drinking water. (**A**) Scheme of treatment with anti-IFN-γ neutralized antibody during late stage of CAC in SHP2$^{CD4-/-}$ mice. (**B**) The body weight of mice was record. (**C**) ELISA analysis of IFN-γ in colonic tissues from WT mice, SHP2$^{CD4-/-}$ mice plus anti-IgG control and SHP2$^{CD4-/-}$ mice plus anti-IFN-γ antibody at day 80. Data are presented as means ± SEM (n = 5). *P < 0.05, **P < 0.01 vs. as indicated.

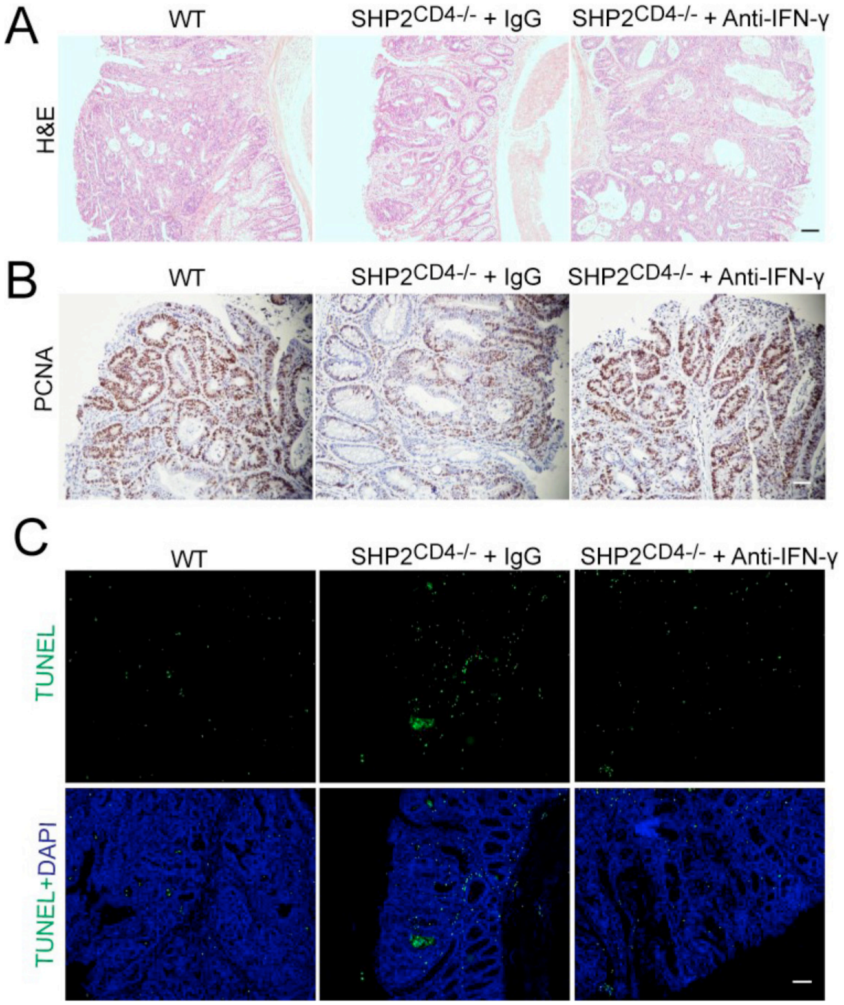

Supplementary Figure S8: IFN-γ neutralization reverses the inhibitory effect of SHP2-deficiency on CAC. WT and SHP2$^{CD4-/-}$ mice were induced with AOM-DSS colitis-associated colon cancer (CAC) model. Mice were injected intraperitoneally with 25 μg anti-IFN-γ neutralized antibody or anti-IgG antibody every 4 days after the last DSS cycle. (**A**) Hematoxylin and eosin (H&E)-stained colonic tissue sections harvested from CAC mice on day 80. Scale bar, 100 μm. (**B**) Immunochemistry analysis of PCNA expression on colonic tissue sections from CAC mice on day 80. Scale bar, 100 μm. (**C**) Apoptosis was analyzed by TUNEL staining in colonic tissues from CAC mice on day 80. Data are representative of three independent experiments.

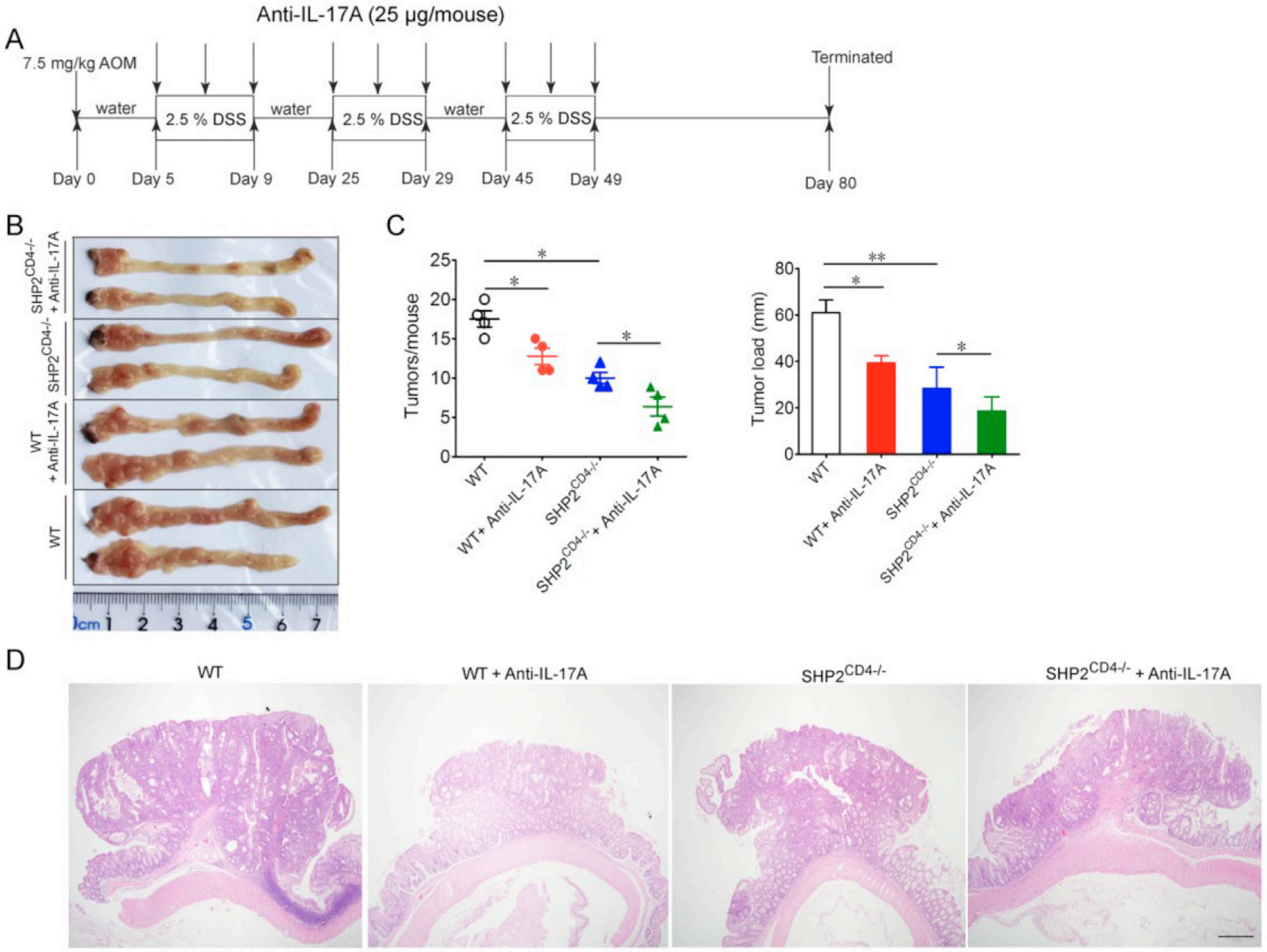

Supplementary Figure S9: IL-17A neutralization reduces tumor growth on CAC mice. (**A**) Scheme of treatment with IL-17A neutralized antibody during CAC in SHP2$^{CD4-/-}$ mice. Mice were injected intraperitoneally (i.p.) with 25 μg anti-IL-17A neutralized antibody or anti-IgG control every 3 days during DSS cycle. (**B**) The inside of the colon was photographed. (**C**) The number of tumors and tumor load were determined. (**D**) Hematoxylin and eosin (H&E)-stained colonic tissue sections harvested from CAC mice on day 80. Data are representative of three independent experiments (mean ± SEM of 5 mice per group in C). *P < 0.05, **P < 0.01.

Supplementary Table S1: Patient information for tissue arrays of human malignant colon cancer tissues of different clinical stages (carcinoma, stages I–IV). See Supplementary_Table_S1
