# Supplementary material for: T lymphocyte SHP2-deficiency triggers anti-tumor immunity to inhibit colitis-associated cancer in mice
Source: Oncotarget. 2016 Dec 7;8(5):7586–97. doi: 10.18632/oncotarget.13812 (PMC5352345; doi:10.18632/oncotarget.13812)
Supplement: Supplementary file 2 [file oncotarget-08-7586-s002.docx]

| **NO** | **sex** | **age** | **organ** | **pathology** | **grade** | **stage** | **tnm** | **type** |
| --- | --- | --- | --- | --- | --- | --- | --- | --- |
| A1 | M | 49 | Colon | Adenocarcinoma | 1 | I | T2N0M0 | Malignant |
| A2 | M | 56 | Colon | Adenocarcinoma | 1 | II | T3N0M0 | Malignant |
| A3 | F | 55 | Colon | Mucinous adenocarcinoma | 1 | III | T3N1M0 | Malignant |
| A4 | F | 56 | Colon | Adenocarcinoma | 1 | II | T3N0M0 | Malignant |
| A5 | F | 38 | Colon | Mucinous adenocarcinoma | 1 | II | T3N0M0 | Malignant |
| A6 | F | 24 | Colon | Mucinous adenocarcinoma | 1 | I | T2N0M0 | Malignant |
| A7 | F | 41 | Colon | Adenocarcinoma | 1 | II | T3N0M0 | Malignant |
| A8 | F | 68 | Colon | Adenocarcinoma | 1 | III | T4N2M0 | Malignant |
| A9 | F | 73 | Colon | Adenocarcinoma | 1 | II | T4N0M0 | Malignant |
| A10 | M | 74 | Colon | Adenocarcinoma | 1 | III | T4N2M0 | Malignant |
| A11 | M | 40 | Colon | Adenocarcinoma | 1 | II | T3N0M0 | Malignant |
| A12 | M | 50 | Colon | Adenocarcinoma | 1 | II | T3N0M0 | Malignant |
| A13 | M | 61 | Colon | Adenocarcinoma | 1 | II | T4N0M0 | Malignant |
| A14 | M | 69 | Colon | Adenocarcinoma (sparse) | 1 | II | T4N0M0 | Malignant |
| A15 | M | 64 | Colon | Mucinous adenocarcinoma | 1 | II | T4N0M0 | Malignant |
| A16 | M | 69 | Colon | Adenocarcinoma | 1 | I | T2N0M0 | Malignant |
| A17 | F | 51 | Colon | Adenocarcinoma | 1 | II | T4N0M0 | Malignant |
| A18 | M | 53 | Colon | Adenocarcinoma | 1 | II | T3N0M0 | Malignant |
| B1 | M | 46 | Colon | Adenocarcinoma | 1 | III | T3N1M0 | Malignant |
| B2 | M | 27 | Colon | Adenocarcinoma | 1 | I | T2N0M0 | Malignant |
| B3 | M | 75 | Colon | Adenocarcinoma | 1 | II | T3N0M0 | Malignant |
| B4 | F | 75 | Colon | Adenocarcinoma | 1 | II | T3N0M0 | Malignant |
| B5 | M | 50 | Colon | Adenocarcinoma | 1 | III | T4N1M0 | Malignant |
| B6 | M | 43 | Colon | Adenocarcinoma | 1 | II | T4N0M0 | Malignant |
| B7 | F | 70 | Colon | Adenocarcinoma | 2 | IV | T4N1M1 | Malignant |
| B8 | M | 59 | Colon | Adenocarcinoma | 1 | II | T4N0M0 | Malignant |
| B9 | M | 70 | Colon | Adenocarcinoma | 1 | III | T3N2M0 | Malignant |
| B10 | M | 45 | Colon | Adenocarcinoma | 1 | II | T3N0M0 | Malignant |
| B11 | M | 30 | Colon | Adenocarcinoma | 1 | I | T1N0M0 | Malignant |
| B12 | M | 46 | Colon | Adenocarcinoma | 1 | III | T3N2M0 | Malignant |
| B13 | F | 44 | Colon | Mucinous adenocarcinoma (sparse) | 1 | II | T3N0M0 | Malignant |
| B14 | M | 50 | Colon | Adenocarcinoma | 1 | II | T3N0M0 | Malignant |
| B15 | F | 40 | Colon | Mucinous adenocarcinoma (sparse) | 1 | II | T3N0M0 | Malignant |
| B16 | M | 56 | Colon | Adenocarcinoma | 1 | III | T3N2M0 | Malignant |
| B17 | M | 51 | Colon | Adenocarcinoma | 1 | III | T2N1M0 | Malignant |
| B18 | F | 68 | Colon | Mucinous adenocarcinoma | 1 | III | T3N1M0 | Malignant |
| C1 | M | 68 | Colon | Mucinous adenocarcinoma | 2 | I | T2N0M0 | Malignant |
| C2 | M | 32 | Colon | Adenocarcinoma | 1 | IV | T4N0M1 | Malignant |
| C3 | M | 46 | Colon | Adenocarcinoma | 1 | II | T3N0M0 | Malignant |
| C4 | M | 64 | Colon | Adenocarcinoma | 1 | II | T4N0M0 | Malignant |
| C5 | M | 58 | Colon | Adenocarcinoma | 1 | III | T4N1M0 | Malignant |
| C6 | F | 73 | Colon | Adenocarcinoma | 1 | II | T4N0M0 | Malignant |
| C7 | M | 45 | Colon | Adenocarcinoma | 1 | I | T2N0M0 | Malignant |
| C8 | M | 54 | Colon | Adenocarcinoma | 2 | II | T3N0M0 | Malignant |
| C9 | F | 51 | Colon | Adenocarcinoma | 1 | IV | T4N1M1 | Malignant |
| C10 | F | 63 | Colon | Adenocarcinoma (smooth muscle and blood vesseltissue) | - | I | T2N0M0 | Malignant |
| C11 | F | 52 | Colon | Adenocarcinoma | 1 | III | T3N1M0 | Malignant |
| C12 | F | 67 | Colon | Adenocarcinoma | 2 | II | T3N0M0 | Malignant |
| C13 | M | 45 | Colon | Adenocarcinoma | 1 | II | T3N0M0 | Malignant |
| C14 | M | 41 | Colon | Adenocarcinoma | 1 | II | T4N0M0 | Malignant |
| C15 | F | 69 | Colon | Adenocarcinoma | 1 | I | T2N0M0 | Malignant |
| C16 | F | 49 | Colon | Mucinous adenocarcinoma | 1 | I | T2N0M0 | Malignant |
| C17 | F | 56 | Colon | Adenocarcinoma | 2 | III | T2N1M0 | Malignant |
| C18 | M | 62 | Colon | Adenocarcinoma | 1 | II | T3N0M0 | Malignant |
| D1 | M | 76 | Colon | Adenocarcinoma (chronic inflammation of mucous membrane) | - | III | T4N2M0 | Malignant |
| D2 | M | 66 | Colon | Adenocarcinoma | 1 | II | T4N0M0 | Malignant |
| D3 | F | 63 | Colon | Adenocarcinoma | 1 | II | T3N0M0 | Malignant |
| D4 | M | 66 | Colon | Adenocarcinoma | 1 | II | T4N0M0 | Malignant |
| D5 | M | 73 | Colon | Mucinous adenocarcinoma | 1 | II | T3N0M0 | Malignant |
| D6 | M | 39 | Colon | Mucinous adenocarcinoma | 1 | II | T3N0M0 | Malignant |
| D7 | F | 74 | Colon | Mucinous adenocarcinoma | 1 | II | T3N0M0 | Malignant |
| D8 | F | 45 | Colon | Mucinous adenocarcinoma | 1 | III | T4N2M0 | Malignant |
| D9 | M | 68 | Colon | Mucinous adenocarcinoma | 1 | II | T3N0M0 | Malignant |
| D10 | F | 67 | Colon | Adenocarcinoma | 2 | I | T2N0M0 | Malignant |
| D11 | M | 73 | Colon | Adenocarcinoma | 2 | II | T4N0M0 | Malignant |
| D12 | M | 50 | Colon | Adenocarcinoma | 2 | II | T4N0M0 | Malignant |
| D13 | F | 51 | Colon | Adenocarcinoma | 2 | IV | T4N0M1 | Malignant |
| D14 | F | 62 | Colon | Adenocarcinoma | 2 | II | T4N0M0 | Malignant |
| D15 | F | 58 | Colon | Adenocarcinoma | 1 | II | T3N1M0 | Malignant |
| D16 | F | 41 | Colon | Adenocarcinoma | 1 | III | T3N1M0 | Malignant |
| D17 | F | 48 | Colon | Adenocarcinoma | 1 | II | T3N0M0 | Malignant |
| D18 | F | 61 | Colon | Adenocarcinoma | 1 | I | T2N0M0 | Malignant |
| E1 | M | 31 | Colon | Adenocarcinoma | 1 | II | T4N0M0 | Malignant |
| E2 | F | 53 | Colon | Adenocarcinoma | 2 | I | T2N0M0 | Malignant |
| E3 | F | 49 | Colon | Adenocarcinoma | 2 | III | T3N1M0 | Malignant |
| E4 | F | 64 | Colon | Adenocarcinoma | 2 | II | T3N0M0 | Malignant |
| E5 | M | 76 | Colon | Adenocarcinoma | 2 | II | T3N0M0 | Malignant |
| E6 | M | 61 | Colon | Adenocarcinoma | 2 | III | T3N1M0 | Malignant |
| E7 | F | 72 | Colon | Adenocarcinoma (sparse) | 2 | III | T3N1M0 | Malignant |
| E8 | M | 65 | Colon | Adenocarcinoma | 2 | II | T3N0M0 | Malignant |
| E9 | M | 41 | Colon | Adenocarcinoma | 2 | III | T4N1M0 | Malignant |
| E10 | F | 69 | Colon | Adenocarcinoma | 2 | II | T3N0M0 | Malignant |
| E11 | M | 62 | Colon | Adenocarcinoma | 2 | II | T3N0M0 | Malignant |
| E12 | F | 48 | Colon | Adenocarcinoma | 2 | III | T4N1M0 | Malignant |
| E13 | F | 70 | Colon | Adenocarcinoma | 2 | I | T3N0M0 | Malignant |
| E14 | F | 78 | Colon | Adenocarcinoma | 2 | III | T3N0M0 | Malignant |
| E15 | F | 47 | Colon | Adenocarcinoma | 2 | II | T3N0M0 | Malignant |
| E16 | F | 66 | Colon | Adenocarcinoma | 2 | II | T4N0M0 | Malignant |
| E17 | M | 64 | Colon | Adenocarcinoma | 2 | III | T3N1M0 | Malignant |
| E18 | M | 62 | Colon | Adenocarcinoma | 2 | II | T3N0M0 | Malignant |
| F1 | F | 55 | Colon | Adenocarcinoma (sparse) | 2 | II | T3N0M0 | Malignant |
| F2 | M | 82 | Colon | Adenocarcinoma (fibrous tissue) | - | II | T4N0M0 | Malignant |
| F3 | M | 80 | Colon | Adenocarcinoma (sparse) | - | III | T4N1M0 | Malignant |
| F4 | M | 59 | Colon | Adenocarcinoma | 1 | II | T4N0M0 | Malignant |
| F5 | M | 53 | Colon | Adenocarcinoma | 2 | II | T4N0M0 | Malignant |
| F6 | M | 72 | Colon | Adenocarcinoma | 2 | II | T4N0M0 | Malignant |
| F7 | M | 46 | Colon | Adenocarcinoma | 2 | II | T4N0M0 | Malignant |
| F8 | F | 69 | Colon | Adenocarcinoma | 2 | III | T3N1M0 | Malignant |
| F9 | F | 58 | Colon | Adenocarcinoma | 2 | II | T4N0M0 | Malignant |
| F10 | F | 54 | Colon | Adenocarcinoma | 2 | II | T4N0M0 | Malignant |
| F11 | M | 73 | Colon | Adenocarcinoma | 2 | II | T3N0M0 | Malignant |
| F12 | M | 71 | Colon | Adenocarcinoma (sparse) | - | II | T3N0M0 | Malignant |
| F13 | M | 45 | Colon | Adenocarcinoma | 2 | III | T4N1M0 | Malignant |
| F14 | F | 62 | Colon | Adenocarcinoma | 2 | III | T4N1M0 | Malignant |
| F15 | F | 52 | Colon | Adenocarcinoma | 2 | III | T3N1M0 | Malignant |
| F16 | M | 69 | Colon | Adenocarcinoma | 2 | II | T3N0M0 | Malignant |
| F17 | M | 70 | Colon | Adenocarcinoma | 2 | I | T2N0M0 | Malignant |
| F18 | F | 79 | Colon | Adenocarcinoma | 2 | II | T4N0M0 | Malignant |
| G1 | M | 40 | Colon | Adenocarcinoma | 2 | III | T4N1M0 | Malignant |
| G2 | M | 42 | Colon | Adenocarcinoma | 2 | III | T3N1M0 | Malignant |
| G3 | M | 40 | Colon | Adenocarcinoma | 2 | II | T4N0M0 | Malignant |
| G4 | F | 67 | Colon | Adenocarcinoma | 2 | II | T3N0M0 | Malignant |
| G5 | M | 53 | Colon | Adenocarcinoma | 1 | II | T3N0M0 | Malignant |
| G6 | M | 41 | Colon | Adenocarcinoma | 2 | II | T3N0M0 | Malignant |
| G7 | M | 41 | Colon | Adenocarcinoma | 1 | II | T4N0M0 | Malignant |
| G8 | F | 46 | Colon | Adenocarcinoma | 1 | II | T3N0M0 | Malignant |
| G9 | F | 60 | Colon | Adenocarcinoma | 1 | III | T4N1M0 | Malignant |
| G10 | F | 70 | Colon | Adenocarcinoma | 2 | II | T3N0M0 | Malignant |
| G11 | F | 26 | Colon | Adenocarcinoma | 2 | II | T3N0M0 | Malignant |
| G12 | M | 34 | Colon | Adenocarcinoma | 1 | II | T3N0M0 | Malignant |
| G13 | M | 62 | Colon | Adenocarcinoma | 1 | II | T4N0M0 | Malignant |
| G14 | M | 69 | Colon | Adenocarcinoma | 1 | I | T2N0M0 | Malignant |
| G15 | M | 49 | Colon | Adenocarcinoma | 2 | II | T3N0M0 | Malignant |
| G16 | M | 69 | Colon | Adenocarcinoma | 2 | II | T3N0M0 | Malignant |
| G17 | M | 71 | Colon | Adenocarcinoma | 2 | II | T4N0M0 | Malignant |
| G18 | F | 49 | Colon | Adenocarcinoma | 2 | II | T3N0M0 | Malignant |
| H1 | F | 53 | Colon | Adenocarcinoma | 2 | II | T3N0M0 | Malignant |
| H2 | M | 72 | Colon | Adenocarcinoma | 2 | I | T2N0M0 | Malignant |
| H3 | M | 50 | Colon | Adenocarcinoma | 2 | II | T3N0M0 | Malignant |
| H4 | F | 53 | Colon | Adenocarcinoma | 2 | II | T3N0M0 | Malignant |
| H5 | F | 58 | Colon | Adenocarcinoma | 2 | IV | T3N0M1 | Malignant |
| H6 | M | 53 | Colon | Adenocarcinoma | 2 | II | T3N0M0 | Malignant |
| H7 | M | 34 | Colon | Adenocarcinoma | 2 | II | T3N0M0 | Malignant |
| H8 | M | 62 | Colon | Adenocarcinoma | 2 | II | T4N0M0 | Malignant |
| H9 | F | 42 | Colon | Adenocarcinoma | 2 | II | T3N0M0 | Malignant |
| H10 | F | 69 | Colon | Adenocarcinoma | 2 | II | T3N0M0 | Malignant |
| H11 | F | 54 | Colon | Adenocarcinoma | 2 | III | T4N1M0 | Malignant |
| H12 | M | 61 | Colon | Adenocarcinoma | 2 | III | T3N2M0 | Malignant |
| H13 | M | 57 | Colon | Mucinous adenocarcinoma | 2 | II | T3N1M0 | Malignant |
| H14 | M | 43 | Colon | Mucinous adenocarcinoma | 2 | I | T2N0M0 | Malignant |
| H15 | M | 74 | Colon | Adenocarcinoma (smooth muscle) | - | III | T3N1M0 | Malignant |
| H16 | M | 51 | Colon | Mucinous adenocarcinoma | 2 | I | T2N0M0 | Malignant |
| H17 | M | 78 | Colon | Adenocarcinoma | 2 | II | T3N0M0 | Malignant |
| H18 | M | 50 | Colon | Adenocarcinoma | 2 | II | T3N0M0 | Malignant |
| I1 | F | 44 | Colon | Mucinous adenocarcinoma | 1 | II | T3N0M0 | Malignant |
| I2 | F | 74 | Colon | Adenocarcinoma (smooth muscle and blood vesseltissue) | - | II | T3N0M0 | Malignant |
| I3 | F | 70 | Colon | Mucinous adenocarcinoma (sparse) | 1 | II | T3N0M0 | Malignant |
| I4 | M | 49 | Colon | Mucinous adenocarcinoma | 2 | II | T3N0M0 | Malignant |
| I5 | F | 48 | Colon | Adenocarcinoma | 2 | IV | T4N0M1 | Malignant |
| I6 | M | 57 | Colon | Adenocarcinoma | 1 | II | T4N0M0 | Malignant |
| I7 | F | 57 | Colon | Adenocarcinoma | 2 | I | T2N0M0 | Malignant |
| I8 | M | 53 | Colon | Adenocarcinoma | 2 | II | T3N0M0 | Malignant |
| I9 | M | 65 | Colon | Adenocarcinoma | 2 | II | T4N0M0 | Malignant |
| I10 | F | 50 | Colon | Adenocarcinoma | 2 | II | T3N0M0 | Malignant |
| I11 | F | 47 | Colon | Adenocarcinoma | 3 | II | T3N0M0 | Malignant |
| I12 | F | 34 | Colon | Adenocarcinoma | 3 | II | T4N0M0 | Malignant |
| I13 | M | 46 | Colon | Adenocarcinoma with necrosis | 3 | II | T4N0M0 | Malignant |
| I14 | F | 47 | Colon | Adenocarcinoma | 3 | II | T3N0M0 | Malignant |
| I15 | M | 64 | Colon | Adenocarcinoma (fibrous and blood vessel tissue) | - | II | T3N0M0 | Malignant |
| I16 | M | 40 | Colon | Adenocarcinoma | 3 | III | T4N2M0 | Malignant |
| I17 | M | 55 | Colon | Adenocarcinoma | 3 | II | T3N0M0 | Malignant |
| I18 | F | 42 | Colon | Adenocarcinoma | 3 | II | T3N0M0 | Malignant |
| J1 | F | 73 | Colon | Adenocarcinoma | 3 | II | T3N0M0 | Malignant |
| J2 | F | 62 | Colon | Adenocarcinoma | 3 | III | T3N1M0 | Malignant |
| J3 | M | 64 | Colon | Adenocarcinoma (sparse) | - | I | T2N0M0 | Malignant |
| J4 | F | 48 | Colon | Adenocarcinoma | 3 | IV | T4N1M1 | Malignant |
| J5 | M | 37 | Colon | Adenocarcinoma | 3 | II | T4N0M0 | Malignant |
| J6 | M | 72 | Colon | Adenocarcinoma | 3 | II | T3N0M0 | Malignant |
| J7 | F | 38 | Colon | Adenocarcinoma | 3 | III | T4N1M0 | Malignant |
| J8 | F | 48 | Colon | Adenocarcinoma | 3 | IV | T4N2M1 | Malignant |
| J9 | M | 65 | Colon | Adenocarcinoma | 3 | III | T3N1M0 | Malignant |
| J10 | M | 53 | Colon | Adenocarcinoma | 3 | IV | T3N2M1 | Malignant |
| J11 | F | 48 | Colon | Adenocarcinoma (sparse) | 3 | II | T3N0M0 | Malignant |
| J12 | F | 73 | Colon | Adenocarcinoma | 3 | III | T4N1M0 | Malignant |
| J13 | M | 38 | Colon | Adenocarcinoma | 3 | II | T4N0M0 | Malignant |
| J14 | M | 48 | Colon | Adenocarcinoma | 3 | IV | T4N2M1 | Malignant |
| J15 | M | 50 | Colon | Adenocarcinoma | 1 | I | T2N0M0 | Malignant |
| J16 | F | 67 | Colon | Adenocarcinoma | 3 | II | T3N0M0 | Malignant |
| J17 | M | 40 | Colon | Adenocarcinoma | 2 | II | T3N0M0 | Malignant |
| J18 | M | 30 | Colon | Adenocarcinoma | 3 | II | T4N0M0 | Malignant |
| K1 | F | 76 | Colon | Adenocarcinoma | 3 | II | T3N0M0 | Malignant |
| K2 | M | 39 | Colon | Adenocarcinoma | 3 | II | T3N0M0 | Malignant |
| K3 | M | 51 | Colon | Adenocarcinoma | 3 | II | T3N0M0 | Malignant |
| K4 | M | 35 | Colon | Adenocarcinoma | 3 | II | T4N0M0 | Malignant |
| K5 | M | 56 | Colon | Signet-ring cell carcinoma | - | III | T4N2M0 | Malignant |
| K6 | F | 70 | Colon | Adenocarcinoma (sparse) | - | III | T3N1M0 | Malignant |
| K7 | M | 24 | Colon | Mucinous adenocarcinoma | 3 | II | T3N0M0 | Malignant |
| K8 | M | 41 | Colon | Adenocarcinoma | 3 | II | T4N0M0 | Malignant |
| K9 | M | 70 | Colon | Adenocarcinoma (smooth muscle) | - | II | T4N0M0 | Malignant |
| K10 | M | 34 | Colon | Mucinous adenocarcinoma | 3 | III | T3N1M0 | Malignant |
| K11 | F | 58 | Colon | Adenocarcinoma (smooth muscle) | - | II | T3N0M0 | Malignant |
| K12 | M | 38 | Colon | Mucinous adenocarcinoma | 3 | II | T3N0M0 | Malignant |
| K13 | M | 46 | Colon | Mucinous adenocarcinoma | 3 | II | T3N0M0 | Malignant |
| K14 | F | 81 | Colon | Mucinous adenocarcinoma | 3 | III | T4N2M0 | Malignant |
| K15 | F | 28 | Colon | Mucinous adenocarcinoma | 3 | III | T4N1M0 | Malignant |
| K16 | M | 42 | Colon | Mucinous adenocarcinoma | 3 | II | T3N0M0 | Malignant |
| K17 | F | 81 | Colon | Mucinous adenocarcinoma | 2 | II | T3N0M0 | Malignant |
| K18 | F | 67 | Colon | Adenocarcinoma (smooth muscle) | - | II | T3N0M0 | Malignant |
| L1 | M | 59 | Colon | Adenocarcinoma | 3 | II | T4N0M0 | Malignant |
| L2 | M | 59 | Colon | Adenocarcinoma | 3 | II | T3N0M0 | Malignant |
| L3 | F | 56 | Colon | Adenocarcinoma | 3 | IV | T4N1M1 | Malignant |
| L4 | M | 68 | Colon | Adenocarcinoma (sparse) | 3 | III | T4N1M0 | Malignant |
| L5 | F | 68 | Colon | Signet-ring cell carcinoma | - | III | T3N1M0 | Malignant |
| L6 | M | 71 | Colon | Signet-ring cell carcinoma | - | II | T4N0M0 | Malignant |
| L7 | F | 37 | Colon | Signet-ring cell carcinoma | - | II | T3N0M0 | Malignant |
| L8 | F | 75 | Colon | Adenocarcinoma | 3 | II | T3N0M0 | Malignant |
| L9 | F | 35 | Colon | Adenocarcinoma | 3 | II | T4N0M0 | Malignant |
| L10 | M | 64 | Colon | Adenocarcinoma | 3 | III | T3N1M0 | Malignant |
| L11 | M | 35 | Colon | Normal colon tissue | - | - | - | Normal |
| L12 | M | 30 | Colon | Normal colon tissue | - | - | - | Normal |
| L13 | F | 21 | Colon | Normal colon tissue | - | - | - | Normal |
| L14 | M | 30 | Colon | Normal colon tissue | - | - | - | Normal |
| L15 | M | 35 | Colon | Normal colon tissue | - | - | - | Normal |
| L16 | M | 30 | Colon | Normal colon tissue | - | - | - | Normal |
| L17 | M | 25 | Colon | Normal colon tissue | - | - | - | Normal |
| L18 | M | 35 | Colon | Normal colon tissue | - | - | - | Normal |
